# Effectiveness of Virtual Reality Interventions on Perioperative Anxiety, Depression, Blood Pressure, and Heart Rate: Systematic Review and Meta-Analysis of Randomized Controlled Trials

**DOI:** 10.2196/81799

**Published:** 2026-05-19

**Authors:** Shijin Wang, Hong Yan, Zhehui Yang, Yan Liu, Tingting Zhang, Yuanyuan Tang, Yuling Luo

**Affiliations:** 1 School of Nursing, Chengdu University of Traditional Chinese Medicine, No. 1166, West Section of Liutai Avenue, Wenjiang District, Chengdu, Sichuan, 611137, China, 86 18980939967

**Keywords:** virtual reality, patients undergoing surgery, anxiety, depression, blood pressure, heart rate, systematic review, meta-analysis

## Abstract

**Background:**

Perioperative stress hinders patient recovery and poses significant challenges for clinical nursing. It triggers a vicious cycle of negative psychological emotions and adverse physiological stress responses. Immersive virtual reality (VR), an innovative nonpharmacological intervention, has been gradually incorporated into perioperative care, showing promise in alleviating patient stress. However, comprehensive evaluations of its multidimensional efficacy remain lacking.

**Objective:**

This study aims to systematically evaluate the dual regulatory effects of VR interventions on both psychological emotions and objective physiological stress in adult patients undergoing perioperative care.

**Methods:**

Following the PRISMA (Preferred Reporting Items for Systematic Reviews and Meta-Analyses) 2020 guidelines, we comprehensively searched 9 electronic databases for randomized controlled trials (RCTs) published from January 2000 to March 2026. Eligible RCTs evaluated VR combined with routine care vs routine care alone in adult patients (≥18 years). The risk of bias was assessed using the Cochrane Risk of Bias 2 tool (Cochrane Collaboration). Meta-analyses were performed using a random-effects model based on the Hartung-Knapp-Sidik-Jonkman method. We reported 95% CIs and 95% prediction intervals (PIs) to interpret clinical heterogeneity and evaluated evidence certainty using the Grading of Recommendations, Assessment, Development, and Evaluation approach.

**Results:**

We included 42 RCTs involving 4648 participants. Compared to routine care, VR significantly reduced anxiety (standardized mean difference −1.17, 95% CI −1.50 to −0.85; 95% PI −3.16 to 0.81) and depression (standardized mean difference −1.26, 95% CI −1.71 to −0.81; 95% PI −2.39 to −0.13). Physiologically, VR effectively decreased systolic blood pressure (mean difference [MD] −5.12, 95% CI −7.21 to −3.03; 95% PI −11.73 to 1.49), diastolic blood pressure (MD −3.45, 95% CI −5.18 to −1.73; 95% PI −8.63 to 1.72), and heart rate (MD −4.45, 95% CI −5.94 to −2.97; 95% PI −10.15 to 1.24). Subgroup analyses revealed that the anxiolytic effect was more pronounced in Asian populations.

**Conclusions:**

VR serves as a safe and effective adjunctive intervention that exhibits a dual regulatory mechanism, simultaneously mitigating psychological distress and stabilizing hemodynamic parameters in perioperative adults. Unlike existing systematic reviews that are predominantly limited to a single psychological metric (eg, anxiety) or focused on pediatric populations, this review integrates both psychological (anxiety and depression) and objective physiological (blood pressure and heart rate) dimensions into a unified evaluation framework. While average benefits are robust, the wide PIs suggest that true effects may vary across individuals due to clinical heterogeneity. Future standardized, large-scale RCTs with strict blinding are warranted to elevate the certainty of evidence.

## Introduction

Patients undergoing perioperative care encounter significant physical and psychological challenges, frequently manifesting complex psychological and physiological responses. Psychologically, patients not only develop anxiety driven by excessive cognitive appraisal of unknown risks, but also frequently experience concurrent depressive moods. Anxiety and depression often coexist in patients undergoing perioperative care, severely impairing their treatment compliance and motivation for recovery [1,2]. Physiologically, negative emotions such as anxiety and depression directly activate the sympathetic-adrenal medullary system, leading to a massive release of catecholamines, thereby triggering physiological stress responses that include accelerated heart rate and abnormal elevations in blood pressure [3]. Such hemodynamic instability not only complicates anesthesia induction but also potentially precipitates adverse cardiovascular and cerebrovascular events, significantly elevating surgical risks [2,3]. Furthermore, severe psychological stress is significantly correlated with various postoperative complications, including delayed wound healing and exacerbated postoperative symptoms such as nausea, vomiting, and intensified pain [4,5]. Patients with higher levels of perioperative psychological stress typically exhibit heightened pain sensitivity [6] and require greater doses of postoperative analgesics. This consequently prolongs hospital stays and may increase the incidence of complications and the risk of mortality [7].

Current interventions for anxiety and stress in patients undergoing perioperative care include pharmacological treatments (eg, benzodiazepines and analgesics) and nonpharmacological therapies (eg, music therapy and aromatherapy) [8-12]. However, pharmacological treatments are associated with adverse effects such as memory impairment and fatigue; moreover, they may induce respiratory and circulatory depression, thereby compromising patient prognosis [13,14]. Regarding nonpharmacological therapies, music therapy is highly susceptible to patient preferences [15], whereas aromatherapy presents challenges in implementing blinding, carries the risk of allergic reactions, and lacks consistent efficacy [16,17]. As a novel nonpharmacological intervention, virtual reality (VR) provides immersive visual and auditory experiences that enable patients to intuitively visualize the surgical procedure or divert their attention. Consequently, it is increasingly emerging as a new trend in perioperative psychological and physiological interventions [18,19].

VR technology uses computers to generate a virtual environment characterized by immersion, interactivity, and real-time feedback [20,21], rendering it a noninvasive and low-risk intervention. Accumulating evidence indicates that VR exerts a dual regulatory mechanism during the perioperative period. Psychologically, research by Malik et al [19] demonstrated that VR effectively interrupts the vicious cycle of anxiety and depression by diminishing patients’ perceived potential environmental threats [22]. Physiologically, studies by Ugras et al [23] revealed that VR serves as a highly efficient distractive modality; it stimulates the parasympathetic nervous system and antagonizes sympathetic overactivity, thereby effectively reducing and stabilizing blood pressure and heart rate in patients undergoing surgery.

In recent years, multiple systematic reviews and meta-analyses have explored the clinical utility of VR during the perioperative period; however, existing studies still exhibit notable limitations. Early meta-analyses indicated that VR could reduce preoperative anxiety in children, whereas its effect on adults remained insignificant [24]. Although recent reviews have corroborated the mitigating effect of VR on adult anxiety, the included studies presented substantial heterogeneity [25]. More importantly, previous reviews were predominantly confined to a single psychological parameter (eg, anxiety) or postoperative pain, seldom systematically incorporating depression—a pivotal psychological factor that profoundly impacts postoperative recovery—into the evaluation. Concurrently, comprehensive meta-analyses of objective physiological metrics (namely, blood pressure and heart rate) triggered by psychological stress remain scarce [26]. Given the intimate pathophysiological interconnections among perioperative anxiety, depression, blood pressure, and heart rate, this review comprehensively synthesizes the effects of VR interventions on both psychological and physiological dimensions in patients undergoing perioperative care by incorporating the latest randomized controlled trials (RCTs).

Against this backdrop, this study aims to comprehensively evaluate the efficacy of VR interventions on psychological emotions and physiological stress in patients undergoing perioperative care through a systematic review and meta-analysis. The specific objectives are as follows: (1) to evaluate the impact of VR interventions on psychological parameters (anxiety and depression) in patients undergoing perioperative care and (2) to analyze the efficacy of VR interventions on objective physiological stress metrics (blood pressure and heart rate). We hypothesize that, compared to routine care, VR interventions can significantly reduce the levels of anxiety and depression in patients undergoing perioperative care while effectively stabilizing blood pressure and heart rate. This review anticipates providing high-level, evidence-based support for health care professionals in selecting safer and more efficacious novel nonpharmacological interventions, thereby improving perioperative outcomes.

## Methods

### Study Design

This review is a systematic review and meta-analysis. The methodology was conducted in accordance with the Cochrane Handbook for Systematic Reviews of Interventions [27]. The reporting of this review fully complies with the PRISMA (Preferred Reporting Items for Systematic Reviews and Meta-Analysis) 2020 statement and its extension checklists [28], while the search process adhered to the PRISMA-S (Preferred Reporting Items for Systematic Reviews and Meta-Analysis–Search) statement for searching [29]. The protocol for this review was prospectively registered in the International Prospective Register of Systematic Reviews (PROSPERO; registration no CRD42025645987), and the study was conducted without any deviations from the preregistered protocol. It should be noted that the primary outcome originally registered was perioperative anxiety. During the conduct of the review, as the understanding of perioperative stress responses evolved and relevant recent literature emerged, we recognized that depression, blood pressure, and heart rate are pathophysiologically interconnected with anxiety and are of comparable clinical importance for perioperative nursing decisions. Consequently, while maintaining the original study design, inclusion criteria, and analytical framework, we expanded the outcome set to include depression, blood pressure, and heart rate as secondary outcomes. All meta-analytic methods, heterogeneity assessments, and effect size syntheses were applied uniformly to the finalized set of outcomes.

### Literature Search Strategy

#### Search Scope and Timeline

A systematic search was conducted across 9 English and Chinese databases, comprising PubMed, Embase (via Ovid), the Cochrane Library (via Wiley), Web of Science, Scopus, CNKI (China National Knowledge Infrastructure), Wanfang Database, VIP Database (China Science and Technology Journal Database), and CBM (Chinese Biomedical Literature Database). The search time frame spanned from January 1, 2000, to March 6, 2026.

#### Development of the Search Strategy

Strictly adhering to the Cochrane Handbook for Systematic Reviews of Interventions, the search strategy was constructed by combining Medical Subject Headings with free-text terms, restricting search fields (eg, title and abstract), and using Boolean operators (AND, OR, and NOT). A comprehensive expansion of core vocabulary with synonyms and related terms was performed to ensure the search was exhaustive and to minimize the risk of missing relevant literature.

#### Search Terms

English search terms: (Virtual Reality OR VR OR virtual reality technology OR immersive virtual technology) AND (perioperative period OR surgical patient OR preoperative OR intraoperative OR postoperative) AND (Anxiety OR Depression OR Blood Pressure OR Heart Rate OR systolic blood pressure OR diastolic blood pressure). Chinese search terms: (虚拟现实 OR VR OR 沉浸式虚拟技术) AND (围术期 OR 术前 OR 术中 OR 术后 OR 手术患者) AND (焦虑 OR 抑郁 OR 血压 OR 心率 OR 收缩压 OR 舒张压).

#### Additional Search

A manual search of reference lists of the included studies, as well as those of related systematic reviews and meta-analyses, was conducted to further identify literature that may have been missed in the electronic database searches. The detailed search results are provided in Multimedia Appendix 1.

To ensure full compliance with the PRISMA-S guidelines, we detail the following search parameters: searches were conducted individually on the native interfaces of each database (eg, Web of Science Core Collection and Cochrane CENTRAL), rather than simultaneously across a multidatabase platform. Built-in database limiters were applied to restrict publication dates, languages (English and Chinese), document types, and study designs (RCTs); however, no externally published or validated search filters (eg, the Cochrane Highly Sensitive Search Strategy) were used. Furthermore, our search strategy was developed de novo by the research team without reusing strategies from prior reviews, and it was not formally peer-reviewed by an independent information specialist. Regarding other sources, we did not systematically browse unindexed online resources, nor did we search clinical trial registries for unpublished data. Additionally, no additional studies were sought by contacting authors or experts (authors were only contacted for missing data from included studies), and no automated search updates or email alerts were used before paper submission. The inclusion and exclusion criteria are provided in Textbox 1.

Textbox 1. **Inclusion and exclusion criteria.**

**Inclusion criteria**:

- Participants: patients aged ≥18 years in the perioperative period (preoperative, intraoperative, or postoperative), without cognitive impairment.
- Interventions: the control group received routine perioperative nursing care, while the experimental group received a virtual reality (VR) intervention combined with routine care.
- Outcome measures: studies including at least one of the following indicators: anxiety scores, depression scores, blood pressure (mm Hg), and heart rate (bpm).
- Study design: randomized controlled trials (RCTs).
- Language: literature published in Chinese or English.

**Exclusion criteria**:

- Patients using antianxiety or antidepressant medications, or those diagnosed with cognitive impairment.
- Studies for which the full text or complete data remained unavailable even after contacting the original authors.
- Incomplete original papers, such as abstracts, conference proceedings, newspaper articles, and dissertations.
- Low-quality literature with severe methodological flaws, data errors, or duplicate publications.

### Literature Screening and Data Extraction

Two reviewers (SW and ZY) independently conducted the initial screening of titles and abstracts, followed by a full-text review, strictly according to the inclusion and exclusion criteria. EndNote (version 21; Clarivate) software was used to remove duplicate records. Any discrepancies arising during the screening process were resolved through discussion or consultation with a third reviewer (HY) to finalize the list of included studies.

The 2 reviewers (SW and ZY) independently extracted data using a standardized data extraction form. The extracted information included basic study information (author, publication year, and country or region), participant characteristics (sample size, age, and type of surgery), intervention details (type of VR device, intervention content, timing of intervention, and duration of intervention), outcome measures (measurement tools, mean, SD, 95% CI, and sample size), and methodological characteristics of the studies (randomization method, implementation of blinding, and information related to risk of bias).

This review confirmed that all participants included in the analysis were mutually independent, with no instances of double-counting. For studies where multiple VR intervention arms shared a single control group, we followed the guidelines of the Cochrane Handbook for Systematic Reviews of Interventions [27] by evenly dividing the control group sample size while keeping the mean and SD unchanged. This approach prevents the inflation of statistical weight caused by double-counting the control group sample, thereby ensuring independence among comparisons.

### Quality Assessment and Grading of Evidence

Two investigators (SW and ZY) independently assessed the risk of bias for the included RCTs using the Cochrane Risk of Bias 2 tool (Cochrane Collaboration) [30]. The assessment domains included the randomization process, deviations from intended interventions, missing outcome data, measurement of the outcome, and selection of the reported result. The evaluation results were presented using a traffic light system (green=low risk, yellow=some concerns, and red=high risk) to generate a risk of bias summary and graph. Any discrepancies were resolved through consensus or by consulting a third investigator (HY).

The quality of evidence for each outcome indicator was evaluated according to the criteria established by the Grading of Recommendations, Assessment, Development, and Evaluation (GRADE) Working Group [31]. The grading dimensions encompassed study limitations, inconsistency, indirectness, imprecision, and publication bias. The certainty of the evidence was accordingly downgraded based on the specific conditions of each dimension. Ultimately, the grading results were presented using the standardized summary of findings table provided by the GRADE Working Group.

### Data Processing and Statistical Analysis

#### Overview

Statistical analyses were performed using RStudio (Posit Software, PBC) with the *meta* and *metafor* packages. A random-effects model was adopted for all outcome measures [32]. Given the unavoidable clinical and methodological heterogeneity across included studies in terms of surgical types, VR devices, intervention contents, and intervention duration, the true effect sizes exhibited a distributional pattern across studies; thus, the random-effects model was more consistent with the study design of this research. For continuous outcomes, the mean difference (MD) with 95% CI was used for outcomes measured with identical tools (blood pressure and heart rate). The standardized mean difference (SMD) with 95% CI was applied for outcomes assessed with different instruments (anxiety and depression). All effect sizes were calculated using the Hartung-Knapp-Sidik-Jonkman method for CI estimation [33], which improves the accuracy and robustness of pooled effect size estimates and reduces the false-positive rate.

#### Heterogeneity Assessment

Cochran Q test (*P*<.10 indicated significant heterogeneity), τ, and τ² were reported. The 95% prediction interval (PI) was computed, and the *I*² statistic was calculated.

#### Subgroup and Sensitivity Analyses

To explore potential sources of heterogeneity, prespecified subgroup analyses were conducted based on geographical region, timing of intervention, and duration of intervention. To assess the robustness of the synthesized results, a leave-one-out sensitivity analysis was performed.

### Bias Assessment

Potential publication bias was evaluated using funnel plots and the Egger linear regression test for the included studies. The trim-and-fill method was used if asymmetry was detected.

### Ethical Considerations

This study is a systematic review and meta-analysis based on previously published literature. As it did not involve primary data collection or direct interaction with human participants or animals, ethical review and approval by an institutional review board, as well as informed consent, were not required.

## Results

### Literature Screening Results

**Figure 1.**
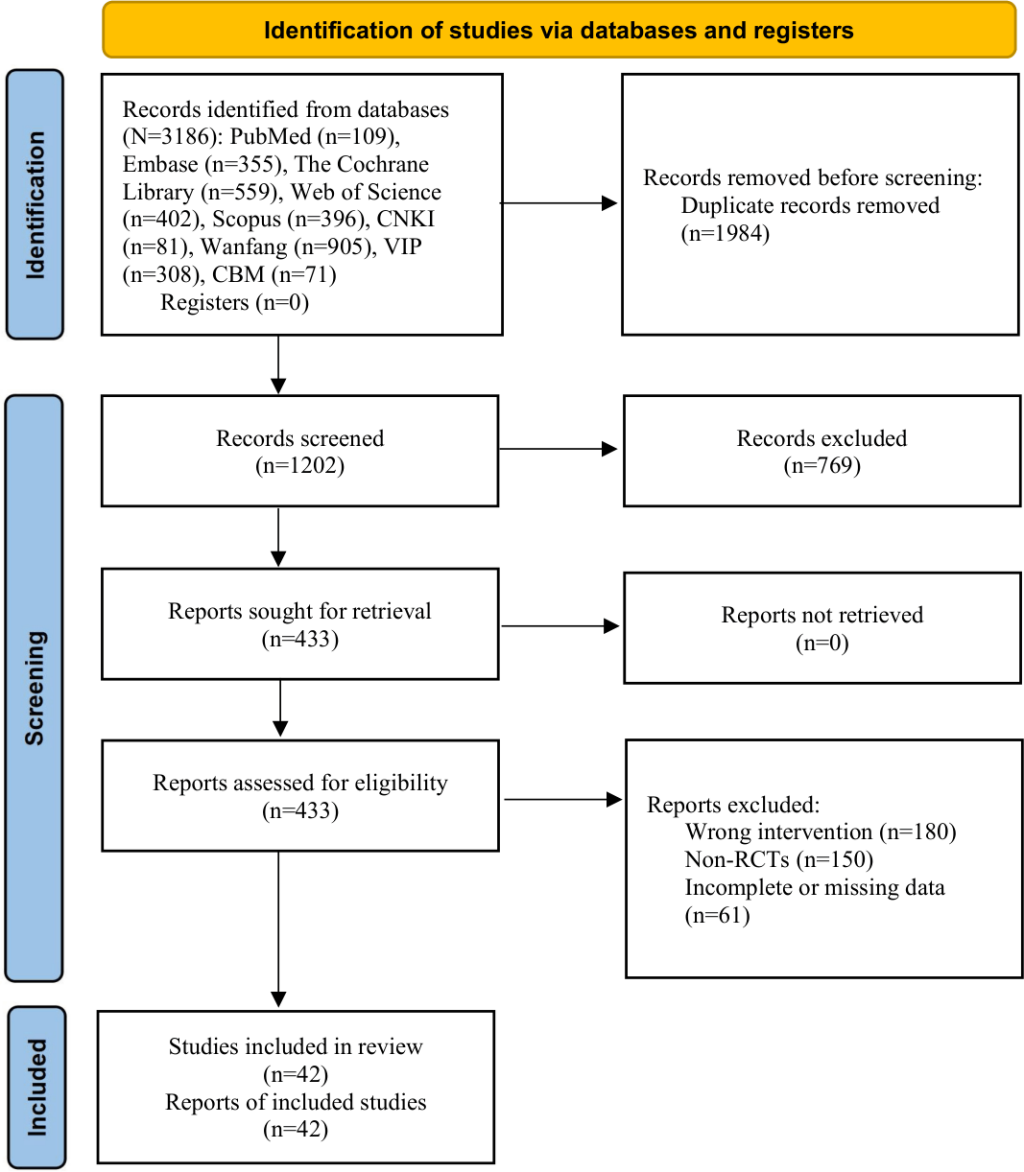
Results of the literature search conducted on March 6, 2026. RCT: randomized controlled trial.

The literature screening process was strictly conducted in accordance with the PRISMA 2020 statement, and the detailed procedure is illustrated in Figure 1. A total of 3186 records were initially retrieved from all databases. After removing 1984 duplicate records using EndNote (version 21) software, 1202 papers remained. Two reviewers (SW and ZY) independently screened the titles and abstracts of these 1202 papers, excluding 769 irrelevant studies due to ineligible participants, inappropriate interventions, or irrelevant outcome measures, leaving 433 papers for full-text evaluation. During the full-text screening phase, an additional 250 studies were excluded for the following reasons: inappropriate interventions (121 studies), non-RCTs (97 studies), and missing data (32 studies). Finally, 42 RCTs were included in this meta-analysis [23,34-74]. Reasons for exclusion of full-text papers are provided in the PRISMA flow diagram in Figure 1.

### Basic Characteristics of Included Studies and Interventions

The 42 included studies were conducted across multiple countries and regions, including China, Turkey, Spain, Iran, France, and others. As shown in Table 1, the studies covered various perioperative populations, including patients undergoing general, orthopedic, cardiovascular, obstetric and gynecological, and oral and maxillofacial surgery. The sample sizes of individual studies ranged from 19 to 423 participants, and all control groups received routine perioperative care.

Regarding intervention measures (Table 2), the presentation and content of VR interventions were highly diverse. Interventions were mainly classified into three categories: (1) natural and relaxing scenes (eg, underwater world, forest, beach, and VR experiences combined with mindful music); (2) perioperative procedural education (eg, first-person simulation of entering the operating room, anesthesia induction, and recovery to reduce fear of the unknown environment); and (3) immersive games or interactive rehabilitation training, with the intervention timing covering the preoperative period (eg, brief 10‐20 minutes intervention in the preoperative waiting area), intraoperative period (eg, continuous use for patients under local or neuraxial anesthesia), and postoperative rehabilitation phase.

**Table 1.** Characteristics of the included studies.

Study and year	Country	Time frame	Surgery type	Study population	Sample size (I^a^/C^b^)	Intervention content		Outcome
						Intervention	Control	
Sun et al (2023) [74]	China	Feb 2022-Jul 2022	Breast biopsy surgery	Patients undergoing breast biopsy (BI-RADS^c^ III-IV; aged 18‐79 years).	46/46	Usual care + VR^d^	Usual care	①^e^
Yang et al (2023) [73]	China	Jun 2021-Jun 2022	CABG^f^	Patients undergoing elective CABG^f^ (aged 18‐80 years).	50/50	Conventional preoperative visit + VR	Conventional preoperative visit	①③④^g^^,^^h^
Yan et al (2024) [72]	China	Jan 2024-May 2024	Cardiac surgery	Patients undergoing their first cardiac surgery under cardiopulmonary bypass (aged ≥18 years).	39/41	Conventional preoperative visit + VR	Conventional preoperative visit	①
Su et al (2025) [70]	China	Jul 2023-Oct 2023	Breast cancer surgery	Female patients with breast cancer undergoing general anesthesia (aged 18‐64 years, BMI 18‐28 kg/m^2^, ASA^i^ I-II, and primary school education or above).	40/40	Routine preoperative education + VR	Routine preoperative education	①④
Ma et al (2023) [71]	China	Nov 2020-Aug 2021	Hip arthroplasty	Patients undergoing hip arthroplasty.	64/64	Routine health education + VR	Routine health education	①②^j^
Liu and Zhu (2023) [68]	China	Feb 2018-Feb 2021	Hepatobiliary surgery	Patients undergoing hepatobiliary surgery (partial hepatectomy, cholecystectomy, or pancreatoduodenectomy; aged 25‐64 years).	54/54	VR-based pain control management platform intervention	Verbal health education	④
Jiang and Yi (2022) [69]	China	Feb 2019-Aug 2020	Primary thyroid surgery	Patients undergoing primary thyroid surgery (aged 18‐65 years; ASA I-II).	44/44	Routine preoperative visit + VR	Routine preoperative visit	①②④
Xue et al (2020) [66]	China	May 2020-Jul 2020	General surgery operations	General surgery patients undergoing general anesthesia (aged 18‐86 years).	50/50	Routine preoperative conversation + first-person perspective VR panoramic video viewing	Routine preoperative conversation	①
Ma et al (2021) [67]	China	Apr 2018-Apr 2020	Intervertebral foramen endoscopic surgery	Patients with lumbar disc herniation undergoing intervertebral foramen surgery (aged 40‐70 years).	49/49	Relaxation training + VR	Relaxation training	④
Liu et al (2023) [64]	China	Jan 2021-Dec 2022	Intra-aortic balloon pump (IABP)	Patients undergoing IABP surgery (aged 25‐80 years).	34/34	Routine early rehabilitation training + VR + bridging exercise	Routine early rehabilitation training	①②
Shang and Li (2021) [65]	China	May 2021-Sep 2021	Intraocular lens implantation	Patients undergoing intraocular lens implantation for age-related cataract (aged 53‐72 years).	40/40	Routine preoperative verbal conversation + VR panoramic video viewing	Routine preoperative verbal conversation	①
Ding (2023) [63]	China	Mar 2019-Jun 2020	Radical hysterectomy	Patients undergoing laparoscopic radical resection of cervical cancer (aged ~52‐54 years).	55/52	Verbal health education + VR	Verbal health education	①②
Xue et al (2024) [62]	China	Jun 2023-Jan 2024	TACE^k^	Patients undergoing TACE for liver cancer (aged 18‐80 years).	38/38	Usual care + VR	Usual care	①
Huang (2023) [61]	China	Oct 2020-Oct 2022	General surgery operations	Young and middle-aged patients with cancer undergoing surgery (aged 25‐60 years).	44/44	Usual care + VR	Usual care	①^e^②^j^
Xu et al (2025) [60]	China	Jan 2023-Jan 2025	Rotator cuff repair	Older patients undergoing rotator cuff repair surgery (aged ≥60 years).	48/48	Rehabilitation training + VR	Rehabilitation training	①
Chen (2022) [59]	China	Nov 2021-Jul 2022	Outpatient surgery	Patients with outpatient surgery (aged 20‐67 years).	423/423	Usual care + VR	Usual care	①③^g^④^h^
Köse et al (2025) [58]	Turkey	Jan 2024-Jun 2024	Per extremity surgery	Patients undergoing upper extremity orthopedic surgery with regional anesthesia (aged ≥18 years).	39/40	Standard perioperative care + VR	Standard perioperative care	①
Yamashita et al (2020) [57]	Japan	Apr 2018-Nov 2018	Oral surgery	Patients undergoing impacted mandibular third molar extraction (aged 20‐49 years).	51/49	Routine surgical treatment + VR	Routine surgical treatment	①
Martínez-Martin et al (2024) [44]	Spain	Sep 2022-Dec 2023	Oral surgery	Adult patients undergoing dental extractions with local anesthesia (aged >18 years).	95/95	Routine surgical treatment + IVR^l^	Routine surgical treatment	①③④
Amiri et al (2023) [43]	Iran	Not specified	Open heart surgery	Patients undergoing open heart surgery (aged 30‐70 years).	30/30	Watched a 360° VR video	Watched the same content video via an iPad	①③④
Keshvari et al (2021) [42]	Iran	Apr 2019-Jul 2019	Coronary artery angiography	Patients undergoing coronary angiography (aged ~51‐52 years).	40/40	Usual care + VR	Usual care	①③④
Singh et al (2024) [56]	India	2023‐2024	Unilateral knee replacement surgery	Patients undergoing unilateral knee replacement under combined spinal epidural anesthesia (aged 18‐65 years and ASA I-III).	33/33	Routine perioperative care + VR	Routine perioperative care	①④
Turan et al (2021) [41]	Turkey	Sep 2017-Jan 2018	Surgery under spinal anesthesia	Patients undergoing surgery under spinal anesthesia (supine position; aged 18‐75 years; and ASA I-II).	50/47	Routine perioperative care + VR	Routine perioperative care	①③④
Almedhesh et al (2022) [40]	Saudi Arabia	Feb 2021-Oct 2021	Cesarean section	Low-risk pregnant women undergoing elective cesarean section under regional anesthesia with normal vision and hearing.	176/175	Routine perioperative care + VR	Routine perioperative care	①③④İnce M, Karaman
İnce and Karaman Özlü (2025) [39]	Turkey	May 2021-Jun 2022	Cesarean section	Patients with cesarean section undergoing spinal anesthesia (aged 18‐65 years).	40/40	Routine perioperative care + VR	Routine perioperative care	①④
Moharam et al (2025) [55]	Egypt	Oct 2023-Aug 2024	Hip arthroplasty	Patients undergoing total hip arthroplasty under spinal anesthesia (aged ≥21 years; ASA I-III).	25/24	Usual care + VR	Usual care	①④
Öz and Demirci (2024) [38]	Turkey	Oct 2022-Mar 2023	Outpatient gynecological procedures	Women undergoing outpatient gynecological procedures without sedation (aged >18 years).	50/50	Usual care + VR	Usual care	①③④
Gül and Yalcinturk (2025) [54]	Turkey	Dec 2023-Feb 2024	Open heart surgery	Patients scheduled for open heart surgery (aged ≥18 years) and no prior cardiovascular surgery.	30/30	Standard preoperative preparation briefing + VR	Standard preoperative preparation briefing	①
Joo et al (2021) [51]	Korea	Dec 2018-Aug 2019	Outpatient surgery	Patients with chronic pain undergoing lumbar sympathetic ganglion block (aged 20‐85 years).	19/19	Routine skin infiltration + VR	Routine skin infiltration	①③④
Erol Akar and Ünver (2025) [53]	Turkey	Feb 2022-Jun 2023	Open-heart surgery	Patients undergoing elective open-heart surgery for the first time (aged ≥18 years).	45/45	Standard preoperative care + VR	Standard preoperative care	①
Baras et al (2025) [52]	France	Not specified	Oral surgery	Patients undergoing extraction of ≥3 wisdom teeth under local anesthesia (aged >14 years).	52/53	Standard oral surgical care + VR	Standard oral surgical care	①
Valls-Ontañón et al (2024) [50]	Spain	Mar 2022-Dec 2022	Oral surgery	Patients undergoing bilateral wisdom tooth extraction under local anesthesia (aged >18 years).	27/27	Usual care + VR	Usual care	①③④
Kwon et al (2023) [49]	Korea	Jun 2019-Dec 2019	Plastic and reconstructive surgery	Patients undergoing general anesthesia (aged >14 years) and no prior surgery experience.	40/40	Preoperative education via VR	Verbal preoperative education	①
Güneş and Sarıtaş (2024) [37]	Turkey	Jun 2020-Aug 2021	Total knee arthroplasty	Patients undergoing total knee arthroplasty (aged >18 years), no prior TKA^m^ history, and pain score ≥4.	65/65	Routine preoperative care via VR	Routine preoperative care	①③④
Ko et al (2024) [47]	China	Jan 2023-Aug 2023	Wound-closure procedures	Adult patients with lacerations requiring suturing in Hong Kong ED^n^ (aged >18 years).	40/40	Standard care + VR	Standard care	①③④
Ugras et al (2023) [23]	Turkey	Jun 2018-May 2019	Colorectal and abdominal wall surgery	Patients undergoing colorectal and abdominal wall surgery (aged 18‐65 years).	43/43	Routine preoperative care + VR	Routine preoperative care	①③④
Rougereau et al (2023) [48]	France	Jun 2020-Sep 2021	Percutaneous hallux valgus surgery	Patients with severe anxiety (STAI^o^ >40) undergoing elective percutaneous hallux valgus surgery (aged >18 years).	30/30	Routine standard care + VR	Routine standard care	①
Oudkerk et al (2022) [46]	Netherlands	Mar 2019-Oct 2020	Percutaneous closure of PFO^p^ or ASD^q^	Patients undergoing percutaneous PFO or ASD closure (aged ≥18 years).	25/25	Routine pre-procedural education + VR	Routine preprocedural education	①
Docimo et al (2026) [45]	Italy	Mar 2024-Mar 2025	Elective day care surgery	Patients undergoing elective surgery (aged 18‐70 years; ASA class ≤ III).	116/116	Standard preoperative care + VR	Standard preoperative care	①
Vogt et al (2021) [36]	Germany	Not specified	Elective surgery with general anesthesia	Patients undergoing elective general anesthesia surgery (aged >18 years; proficient in German).	67/67	Standard care + VR	Standard care	①
Gong et al (2025) [35]	China	Dec 2022-Dec 2024	PCI^r^	Patients after coronary intervention for CHD^s^ (NYHA^t^ class II/III).	46/46	Routine standard care + VR	Routine standard care	①②
Shen et al (2022) [34]	China	Jan 2020-Dec 2020	Surgery for head and neck cancer	Patients with emotional disorders after head and neck cancer surgery (aged 18‐75 years).	38/38	VR sand-play therapy	Routine psychological nursing and health education	①②

a I: intervention group.

b C: control group.

c BI-RADS: Breast Imaging Reporting and Data System.

d VR: virtual reality.

e ①Anxiety.

f CABG: elective coronary artery bypass grafting.

g ③Blood pressure.

h ④Heart rate.

i ASA: American Society of Anesthesiologists.

j ②Depression.

k TACE: transcatheter arterial chemoembolization.

l IVR: immersive virtual reality.

m TKA: total knee arthroplasty.

n ED: emergency department.

o STAI: State-Trait Anxiety Inventory.

p PFO: patent foramen ovale.

q ASD: atrial septal defect.

r PCI: percutaneous coronary intervention.

s CHD: coronary heart disease.

t NYHA: New York Heart Association.

**Table 2.** Detailed interventions in the intervention groups of included studies.

Authors and year	Equipment and systems	Intervention period	Concrete content
Sun et al 2023 [74]	Pico Neo 3 headset and Huawei FreeBuds Pro headphones	Preoperative (15 minutes) and intraoperative (30 minutes)	“Immersive Mindfulness Travel System”: 360 travel videos with guided mindfulness audio for meditation and relaxation during surgery.
Yang et al (2023) [73]	NOLO Sonic VR^a^ equipment	Single 6-minute session 1-day preoperatively	Compiled VR video with departmental introduction, surgery and anesthesia methods, 3D animations of the procedure, and soft background music.
Yan et al (2024) [72]	VR equipment	Single 10-minute bedside session 1-day preoperatively	VR video introducing the ICU^b^ environment, medical team, postoperative tubes, coping methods, breathing exercises, sleep guidance, and rehabilitation.
Su et al (2025) [70]	Insta360 ONE RS camera and Pico Neo 3 headset	Single session 1-day preoperatively	First-person VR video of the entire perioperative journey: preoperative preparation, anesthesia induction, awakening and extubating, and return to ward.
Ma et al (2023) [71]	VR all-in-one headset	During hospitalization (2‐4 times/day)	Preoperative: 360° OR^c^ photos and first-person VR videos. Postoperative: interactive VR rehabilitation games (eg, kitchen and climbing) and exercise videos.
Liu and Zhu (2023) [68]	VR equipment	During postoperative recovery	Patients used a VR pain platform for game-based training on pain perception, pathology, emotions, and attention.
Jiang and Yi (2022) [69]	VR head-mounted display	Single 6-minute session 1 day preoperatively	VR video showing the entire perioperative process: entering the OR, anesthesia induction and maintenance, surgery, and PACU^d^ awakening.
Xue et al (2020) [66]	Obsidian R professional VR camera (6 fisheye lenses)	Single session after preoperative discussion	First-person 360° VR video of the OR journey: triple verification, anesthesia induction, and postoperative awakening process.
Ma et al (2021) [67]	Mobile phone in VR holder	Single 5-minute session before anesthesia	Patients chose and watched relaxing simulated scenarios (eg, “Ice and Snow World” and “Ocean World”) to distract and relax.
Liu et al (2023) [64]	VR equipment	During postoperative recovery	VR-based pain management platform using game-like tasks to train pain perception, pathology, emotions, and attention.
Shang and Li (2021) [65]	Professional VR camera	Single session after preoperative discussion	VR panoramic video of cataract surgery preparation and simulation (triple check, instruments, and operation) to explain the procedure.
Ding (2023) [63]	Professional VR camera (6 fisheye lenses)	Single session before surgery	360° first-person video of the entire surgery process (preoperative checks, anesthesia induction, and awakening) to familiarize patients.
Xue et al (2024) [62]	Hangzhou Xinqing Technology VR device (all-in-one system with 3D video headset, music, and headphones)	During the entire TACE^e^ procedure	Patients chose from 360° relaxing scenes (eg, “Seaside Wind Bells,” “Snowy House,” and “Mountain Waterfall”) and mindfulness scenes for distraction during the awake procedure.
Huang (2023) [61]	Head-mounted display system	From before surgery until the end of the procedure (for young and middle-aged patients with cancer).	Patients experienced VR environments (eg, art museum and deep sea) and games (eg, racing) combined with psychological nursing to distract them from illness and reduce anxiety.
Xu et al (2025) [60]	HTC Vive Focus 3 system (headset, controllers, and tracking system)	3-month rehabilitation program postrotator cuff repair: 2‐3 times/week for 40 minutes, starting post discharge.	VR-based functional training using interactive games (virtual dance, spatial painting, and simulated daily tasks) to guide shoulder exercises and monitor movement; included a calming VR concert for emotional relaxation.
Chen (2022) [59]	VR equipment	Preoperatively as part of the nursing intervention for outpatient surgery.	Patients experienced a first-person VR simulation of the perioperative process (preoperative checks and anesthesia induction) combined with traditional Chinese emotional therapy (“five notes” music therapy and distraction activities).
Köse et al (2025) [58]	Oculus Quest 2 VR headsets and Sony MDRZX110APB wired headphones	Preoperatively during brachial plexus block placement and throughout the intraoperative period for upper extremity surgery.	Patients were immersed in a nature-themed VR environment (forest, snow, and beach) with calming nature sounds for up to 1 hour 46 minutes (replayed if needed).
Yamashita et al (2020) [57]	Oculus Rift CV1^f^ HMD^g^ and custom relaxation VR software	During the entire impacted mandibular third molar extraction procedure under local anesthesia.	Patients watched a custom VR presentation of a large film screen in relaxing settings (cinema, beach, or garden) showing calm nature scenes (sea, rivers, and animals) to induce relaxation.
Martínez-Martin et al (2024) [44]	Shinecon VR glasses (3D)	For a 20-minute session preoperatively in the waiting room and continuously during the entire dental extraction procedure.	Patients experienced immersive 360° images of the ocean floor with relaxing sounds, providing visual and auditory isolation.
Amiri et al (2023) [43]	TSCO VR glasses (model TVR 568)	Single session the day before open heart surgery	Patients watched a 4-minute 35-second 360° educational VR video showing the operating room environment, equipment, and process, to familiarize them with the procedure.
Keshvari et al (2021) [42]	Remix VR video headset (360°), Huawei phone, and headphones	Single 5-minute session, 10 minutes before coronary angiography.	Patients viewed a 5-minute 360° video of natural scenes (beach, mountains, and waterfall) with soft music and nature sounds for distraction.
Singh et al (2024) [56]	IRUSU MONSTER VR headset and Sony WH-1000XM4 noise-canceling headphones	From 1-hour preoperatively, paused for transfer, then continuously intraoperatively (under CSE^h^ anesthesia) until the end of surgery.	Patients watched a self-chosen video via a VR headset, combined with music delivered via noise-canceling headphones for distraction and relaxation during the procedure.
Turan et al (2021) [41]	BOBO VR Z4 glasses	During the entire surgical procedure performed under spinal anesthesia, starting after the block.	Patients watched a movie via VR glasses to provide visual distraction and limit exposure to the operating room environment.
Almedhesh et al (2022) [40]	Oculus Rift S PC-powered VR headset	Immediately after regional anesthesia until completion of skin suturing during cesarean section.	Patients chose to watch 3D natural landscapes with either calm Quran recitation or relaxing music for distraction during the surgery.
İnce and Karaman Özlü (2025) [39]	Samsung Gear VR glasses	During the entire cesarean section procedure under spinal anesthesia (approximately 20‐25 minutes).	Patients watched relaxing videos with a music background (nature, seaside, and submarine images) to provide distraction and reduce anxiety.
Moharam et al (2025) [55]	VR glasses with an audio headset	For a 15-minute preoperative session and continuous intraoperative use during the total hip arthroplasty procedure.	Patients were immersed in a serene environment with nature scenes and soft music to induce relaxation and isolate them from the operating room.
Öz and Demirci (2024) [38]	VR glasses	During the entire outpatient gynecological procedure.	Patients watched a preferred video (forest or sea view) as a distraction method during the procedure.
Gül and Yalcinturk (2025) [54]	VR glasses	Single 15-minute session the night before surgery (10 PM-12 AM).	Patients watched a 15-minute nature-based video (birds chirping, water flowing) in a quiet, calm environment to induce relaxation.
Joo et al (2021) [51]	Samsung Gear HMD on Galaxy 7.0 device and commercial VR hypnosis program (NUVO)	Thirty-minute session during a fluoroscopy-guided lumbar sympathetic ganglion block, starting after group allocation.	Patients in a prone position experienced a VR hypnosis program with a seashore view and Korean narration, designed to induce relaxation during the procedure.
Erol Akar et al (2025) [53]	Samsung VR headset compatible with a smartphone	Single 6.1-minute session on the morning of surgery before transferring to the operating theater.	Patients watched a 360° video titled “Virtual Nature 360°” featuring natural sounds (birds, water, and wind) in a quiet, distraction-free environment.
Baras et al (2025) [52]	RELAXVR glasses and headphones	From the beginning to the end of the tooth extraction procedure.	Patients chose 5 relaxing themes (eg, nature, scuba diving, and animated film) for a full audiovisual immersive experience to reduce anxiety.
Valls-Ontañón et al (2024) [50]	Pico Interactive HMD and external tablet with VR Pharma software	During the entire surgical procedure for one side of the bilateral wisdom tooth extraction.	Patients immersed in relaxing VR content (“Ocean Breeze” or “Crystal Serenity”) to distract from the surgical environment (surgeon-rated comfort).
Kwon et al (2023) [49]	PICO G2 HMD and custom 360° 3D video	Single 11-minute session, the day before surgery, in a private counseling room.	A 360° VR education video showing the step-by-step process of the surgical journey: ward preparation, removal of personal items, transport, and events in the operating room (performed by actual hospital staff).
Günes and Sarıtaş (2024) [37]	VR goggles and a preloaded relaxing video	Single 20-minute session preoperatively in the patient’s room.	Patients watch a 20-minute video of natural scenery (sea, forest, waterfall, and animals) to divert attention from harmful stimuli and promote relaxation.
Ko et al (2024) [47]	VIVA Focus goggles and custom video selection based on patient preference	Single session before local anesthesia and during the procedure.	Patients select and view an immersive 3D video (eg, landscape and ocean) with background music to distract them from the sights, sounds, and sensations of the suturing procedure.
Ugras et al (2023) [23]	VR BOX 2 headset, headphones, and a mobile phone	Single 10-minute session, during transfer to and while waiting in the preoperative holding area.	Patients watch a relaxing video (content not specified but implied to be distracting) via a VR headset to divert attention from the clinical environment and procedure.
Rougereau et al (2023) [48]	Oculus Go and VR hypnosis application	Single 10-minute session, preoperatively in a dedicated room.	A self-guided VR hypnosis session; patients choose the voice (male and female), landscape (sea, beach, and forest), and musical style to induce relaxation and reduce anxiety before surgery.
Oudkerk et al (2022) [46]	Oculus Go and custom 360° VR film	Single 5-minute session immediately after the outpatient consultation (with option to rewatch at home via YouTube [Google LLC]).	A 360° immersive film introducing the care team and providing a virtual walkthrough of the procedure day, including visits to the ward, catheterization laboratory, and recovery room, along with a 3D visualization of the closure procedure. The patient’s partner could also view the film simultaneously.
Docimo et al (2026) [45]	Meta Quest 2 and the Customs in-house application	Single 60-minute session, starting in the surgical ward before entering the operating block.	Patients choose between a documentary, a movie, or a music concert for immersive entertainment, with the aim of providing distraction from the anxiety-provoking preoperative environment.
Vogt et al (2021) [36]	Oculus Go stand-alone VR and custom 360° 3D video	Single 6-minute 28-second session, after the anesthesia interview.	A 360° virtual tour of the perioperative process from a third-person perspective, including the evening before surgery, morning medication, transport to the holding area, the safety checklist conversation, and finally the operating room with anesthesia induction scenes.
Gong et al (2025) [35]	Pico Neo 3 and patient monitor	Three months, starting postoperatively when stable, includes inpatient and continued home-based training.	Upper limb coordination games (eg, reaching and grasping), aerobic exercise in virtual scenes (eg, rowing, cycling, and Tai Chi), and relaxation and meditation in nature scenes.
Shen et al (2022) [34]	VR glasses, voice system, and PC^i^; custom VR sand-play system	13 sessions total: 1 on discharge day, then weekly for 3 months (60 minutes each).	Patients create sand paintings in a virtual, protected environment. A virtual counselor guides them to explore unconscious thoughts and traumas, fostering self-acceptance and healing. The intervention follows themes of injury, transformation, and healing.

a VR: virtual reality.

b ICU: intensive care unit.

c OR: operating room.

d PACU: postanesthesia care unit.

e TACE: transcatheter arterial chemoembolization.

f CV1: consumer version 1.

g HMD: head‑mounted display.

h CSE: combined spinal‑epidural (anesthesia).

i PC: personal computer.

### Risk of Bias Assessment and Quality of Evidence

**Figure 2.**
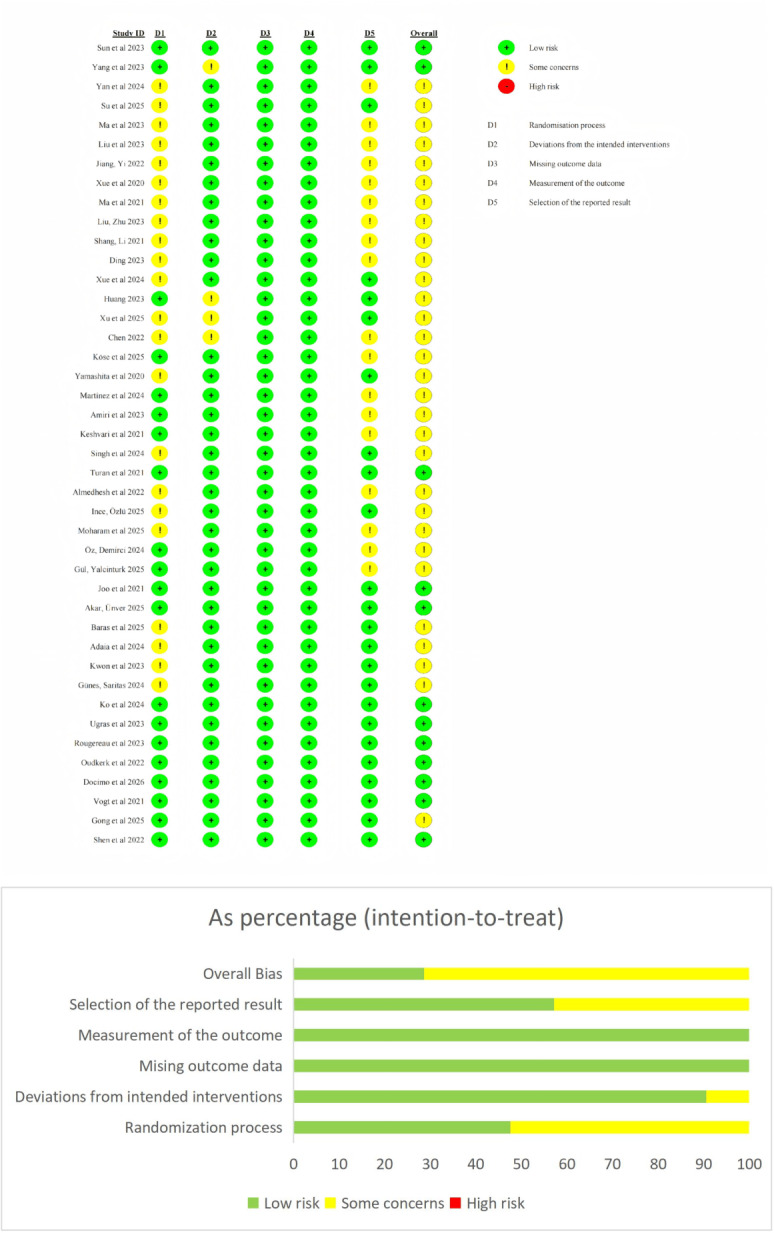
Risk of bias assessment of included randomized controlled trials using the Cochrane Risk of Bias 2 tool [23,34-74].

Two reviewers (SW and ZY) independently assessed the risk of bias for the 42 RCTs using the Risk of Bias 2 tool. Overall, the included studies exhibited favorable methodological quality, and no study was rated as having a high overall risk of bias. The risk-of-bias plot (traffic light plot, Figure 2) revealed that several studies [23,34-74] were rated as having some concerns in the domains of the randomization process (Domain 1) and deviations from intended interventions (Domain 2). The main reasons included insufficient reporting of the specific methods used to conceal the random sequence and the difficulty in achieving complete blinding of participants and personnel due to the inherent nature of VR interventions. The GRADE quality-of-evidence ratings for each outcome measure are summarized in Table 3.

**Table 3.** Grading of Recommendations, Assessment, Development, and Evaluation (GRADE) summary of findings table.

Outcome	Certainty assessment							No of patients		Effect		Certainty	Importance
	No of studies	Study design	Risk of bias	Inconsistency	Indirectness	Imprecision	Other considerations	VR^a^	Routine care	Relative (95% CI)	Absolute (95% CI)		
Perioperative anxiety (assessed using SAS^b^, STAI^c^, HAMA^d^, and VAS^e^; range 25-100)	40	Randomized trials	Not serious	Serious^f^	Not serious	Not serious	None	2327	2321	—^n^	SMD^h^ 1.18 (1.51-0.85)	⨁⨁⨁◯ Moderate^f^	Critical
Perioperative systolic blood pressure (mm Hg; range 60-200)	13	Randomized trials	Not serious	Serious^f^	Not serious	Not serious	None	1108	1104	—	MD^i^ 5.12 (7.21-3.03)	⨁⨁⨁◯ Moderate^f^	Important
Perioperative diastolic blood pressure (mm Hg; range 40-20)	12	Randomized trials	Not serious	Serious^f^	Not serious	Not serious	None	1089	1085	—	MD^i^ 3.45 (5.18-1.73)	⨁⨁⨁◯ Moderate^f^	Important
Perioperative heart rate (bpm; range 40-180)	20	Randomized trials	Not serious	Serious^f^	Not serious	Not serious	None	1393	1388	—	MD 4.45 (5.94-2.97)	⨁⨁⨁◯ Moderate^f^	Important
Perioperative depression (SDS^j^; range 25-100)	7	Randomized trials	Not serious	Serious^f^	Not serious	Not serious	Publication bias strongly suspected	325	322	—	SMD 1.26 (1.72-0.81)	⨁⨁◯◯ Low^f^^,k^	Important

a VR: virtual reality.

b SAS: Self-Rating Anxiety Scale.

c STAI: State-Trait Anxiety Inventory.

d HAMA: Hamilton Anxiety Scale.

e VAS: Visual Analogue Scale.

f The heterogeneity was extremely high, which could not be fully explained by subgroup analysis.

g Not available.

h SMD: standardized mean difference.

i MD: mean difference.

j SDS: Self-Rating Depression Scale.

k Publication bias was suggested by funnel plot asymmetry, but no missing studies were required by trim-and-fill analysis, indicating no significant publication bias. We downgraded the evidence by 1 level due to potential publication bias.

### Results of Meta-Analysis

#### Anxiety

##### Overview

**Figure 3.**
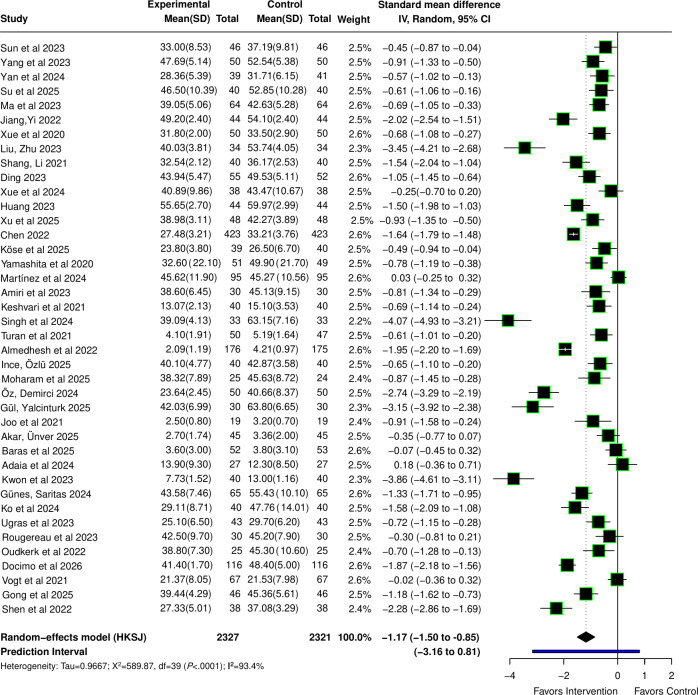
Forest plot for the standardized mean difference (Hedges *g*) of the effect of virtual reality (VR) compared with conventional interventions on anxiety scores in patients undergoing perioperative care; lower anxiety score indicates less anxiety [23,34-66,69-74].

Forty studies [23,34-66,69-74] reported the effect of VR intervention on anxiety in patients undergoing perioperative care, with a total sample size of 4648 participants. The pooled results demonstrated that anxiety levels in the VR group were significantly lower than those in the conventional care group (SMD −1.17, 95% CI −1.50 to −0.85; *P*<.001). Heterogeneity analysis indicated substantial heterogeneity (Q=589.87; *P*<.001; τ=0.967; τ²=0.935; *I*²=93.4%). The computed 95% PI was −3.16 to 0.81, which crossed the line of no effect (0). This suggests that although the overall pooled effect supports the anxiolytic effect of VR, VR intervention may not significantly reduce individual anxiety levels in certain specific clinical settings in the future, highlighting the impact of heterogeneity in intervention protocols and populations on the actual therapeutic effect. The forest plot is shown in Figure 3 [23,34-66,69-74].

To explore the sources of heterogeneity, prespecified subgroup analyses were performed in this review (Table 4).

**Table 4.** Subgroup analysis of anxiety scores in patients undergoing perioperative care receiving virtual reality intervention, stratified by region, timing of intervention, and duration of intervention.

Subgroup	N	SMD^a^ (95% CI)	Prediction intervals	τ		*τ* ^*2*^	Q_M_	*I*^2^ (%)
Region (between-subgroup difference: Q_M_ (df)=9.34 (1); *P*<.05)								
Asia	31	−1.36 (−1.73 to −0.99)	−2.77 to 0.06	0.73		0.53	381.11	92.1
Other	9	−0.49 (−0.99 to 0.01)	−1.97 to 0.99	0.74		0.55	111.85	92.8
Timing of intervention (between-subgroup difference: Q_M_ (df)=9.64 (3); *P*=.02)								
Preoperative	25	−1.12 (−1.51 to −0.72)	−2.67 to 0.44	0.76		0.58	343.69	93
Intraoperative	4	−0.68 (−0.81 to −0.54)	−0.70 to −0.65	0.00		0.00	0.39	0
Postoperative	5	−1.65 (−3.06 to −0.25)	−4.87 to 1.56	0.89		0.78	53.72	92.6
Preoperative + Postoperative	6	−1.32 (−2.88 to 0.25)	−5.10 to 2.47	1.14		1.31	173.95	97.1
Duration of intervention (between-subgroup difference: Q_M_ (df)=4.18 (2); *P*=.12)								
Short-term	3	−2.24 (−5.92 to 1.44)	−11.80 to 7.33	1.31		1.71	46.94	95.7
Medium-term	30	−0.96 (−1.30 to −0.62)	−2.56 to 0.64	0.75		0.56	423.37	93.2
Long-term	7	−1.60 (−2.53 to −0.67)	−3.80 to 0.60	0.79		0.63	75.75	92.1

a SMD: standardized mean difference.

##### Region

Asian region [34,35,59-66,69-74]: SMD −1.36 (95% CI −1.73 to −0.99; 95% PI −2.77 to 0.06). Other regions [36,44-46,48,50,52,55,57]: SMD −0.49 (95% CI −0.99 to 0.01; 95% PI −1.97 to 0.99). The between-subgroup difference was statistically significant (Q=9.34; *P*<.05), indicating that the anxiety-reducing effect of VR intervention may be more pronounced in Asian populations.

##### Timing of Intervention

Intraoperative [39,41-43], postoperative [34,35,60,66,68], preoperative [23,36-38,45-55,57-59,63,65,69-71,73,74], and preoperative + postoperative [40,44,56,61,62,74] interventions all effectively reduced anxiety. Among them, the intraoperative subgroup showed extremely low heterogeneity (*I*²=0%), with SMD −0.68 (95% CI −0.81 to −0.54). Significant differences were observed among timing subgroups (Q=9.64; *P*=.02).

##### Duration of Intervention

VR intervention significantly alleviated anxiety regardless of duration: short duration (≤10 minutes) [49,69,73], medium duration (11‐30 minutes) [23,36-44,46-48,50-59,63,65,66,70-72,74], and long duration (>30 minutes) [34,35,45,60-62,64]. No significant between-subgroup difference was detected (Q=4.18; *P*=.12), suggesting that VR interventions of varying durations provide stable clinical benefits.

### Depression

**Figure 4.**
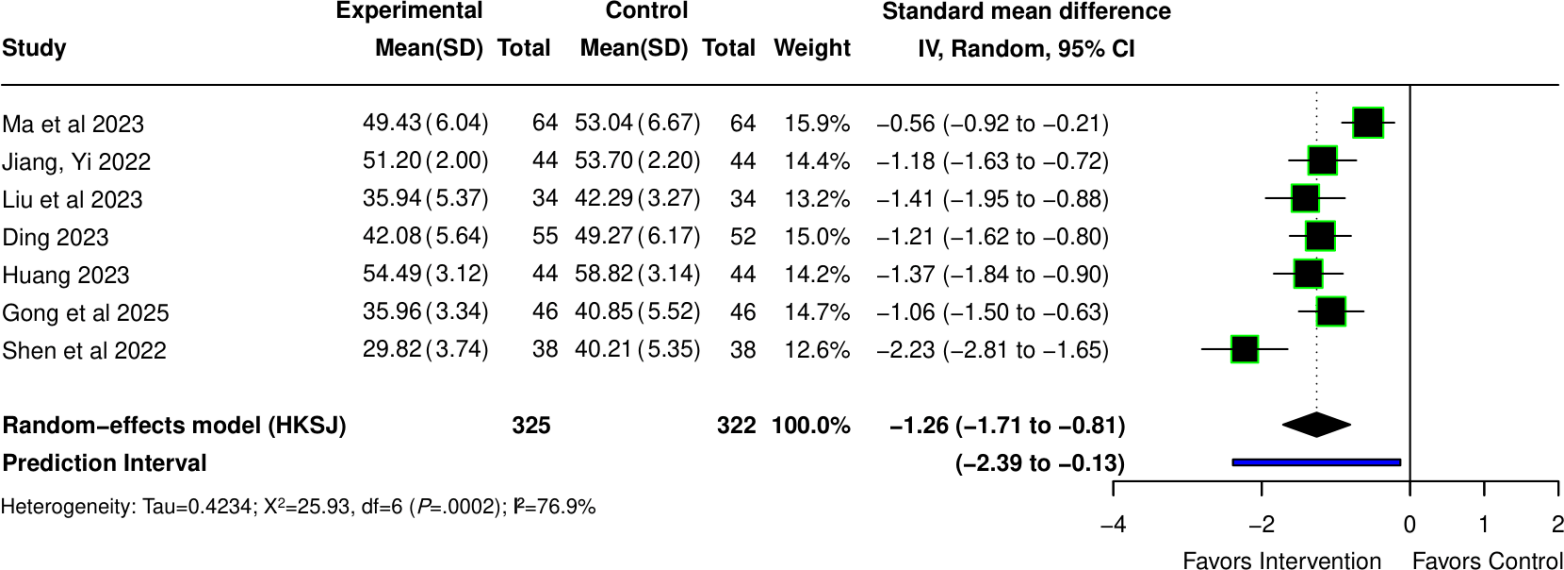
Forest plot for the standardized mean difference (Hedges *g*) of the effect of virtual reality (VR) compared with conventional interventions on depression scores in patients undergoing perioperative care; lower depression scores indicate less depression [34,35,61,63,64,69,71].

Seven studies [34,35,61,63,64,69,71] reported the effect of VR on depression in patients undergoing perioperative care, with a total sample size of 647 participants. The pooled effect size indicated that VR significantly reduced depression scores (SMD −1.26, 95% CI −1.71 to −0.81; *P*<.001). Significant heterogeneity was observed across studies (Q=25.93; *P*<.001; τ=0.423; τ²=0.179; *I*²=76.9%), and the 95% PI was −2.39 to −0.13. The forest plot is provided in Figure 4 [34,35,61,63,64,69,71].

### Blood Pressure

#### Overview

A total of 13 studies [23,37,38,40-44,47,50,51,59,74] and 12 studies [23,37,38,40-44,47,50,59,74] reported the effects of VR on systolic blood pressure (SBP; 2212 patients) and diastolic blood pressure (DBP; 2174 patients) in patients undergoing perioperative care, respectively.

#### SBP Outcomes

SBP in the VR group was significantly lower than that in the control group (MD −5.12, 95% CI −7.21 to −3.03; *P*<.001). Significant heterogeneity was observed (τ=2.839; τ²=8.057; *I*²=69.4%), with a 95% PI of −11.73 to 1.49.

#### DBP Outcomes

**Figure 5.**
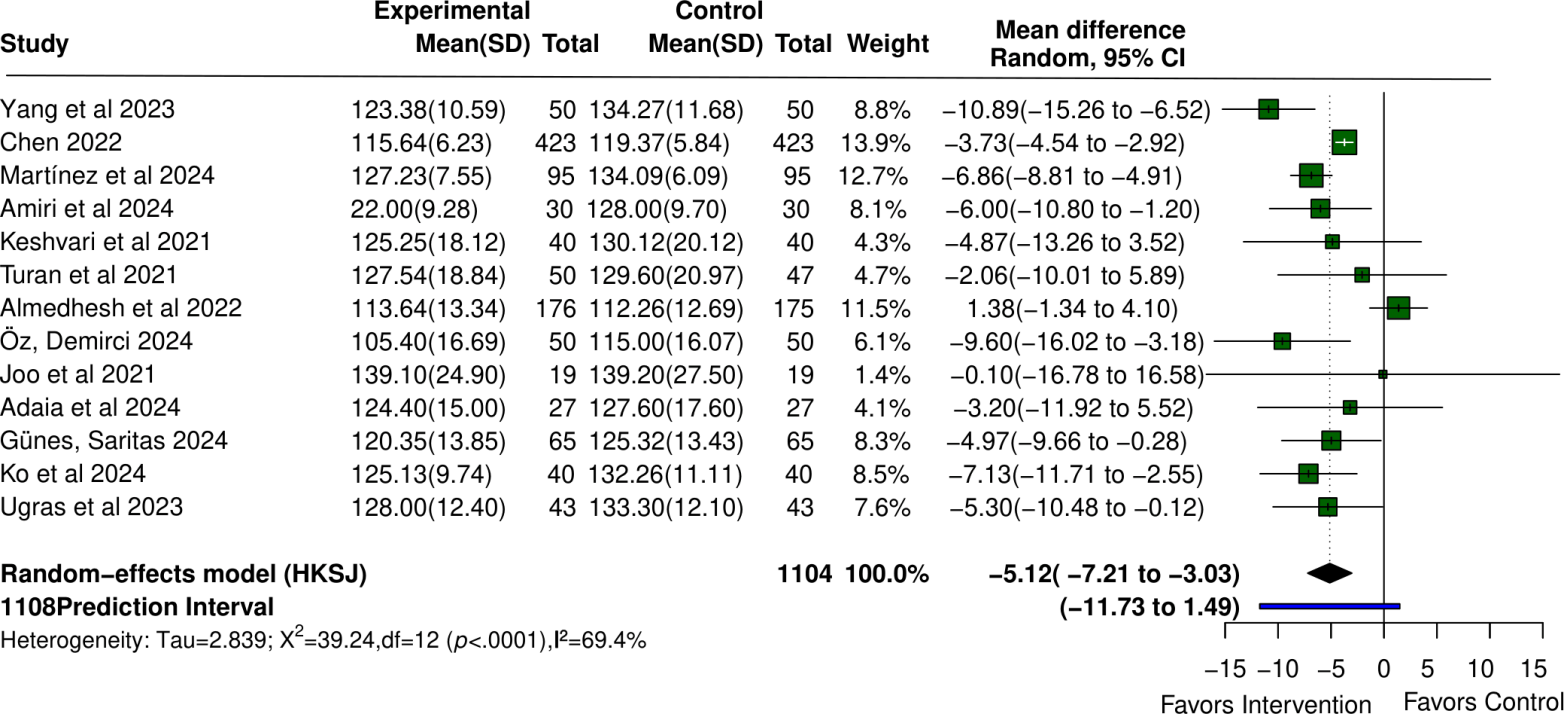
Forest plot of the mean difference for the effect of virtual reality (VR) on systolic blood pressure in patients undergoing perioperative care compared with control [23,37,38,40-44,47,50,51,59,74].

**Figure 6.**
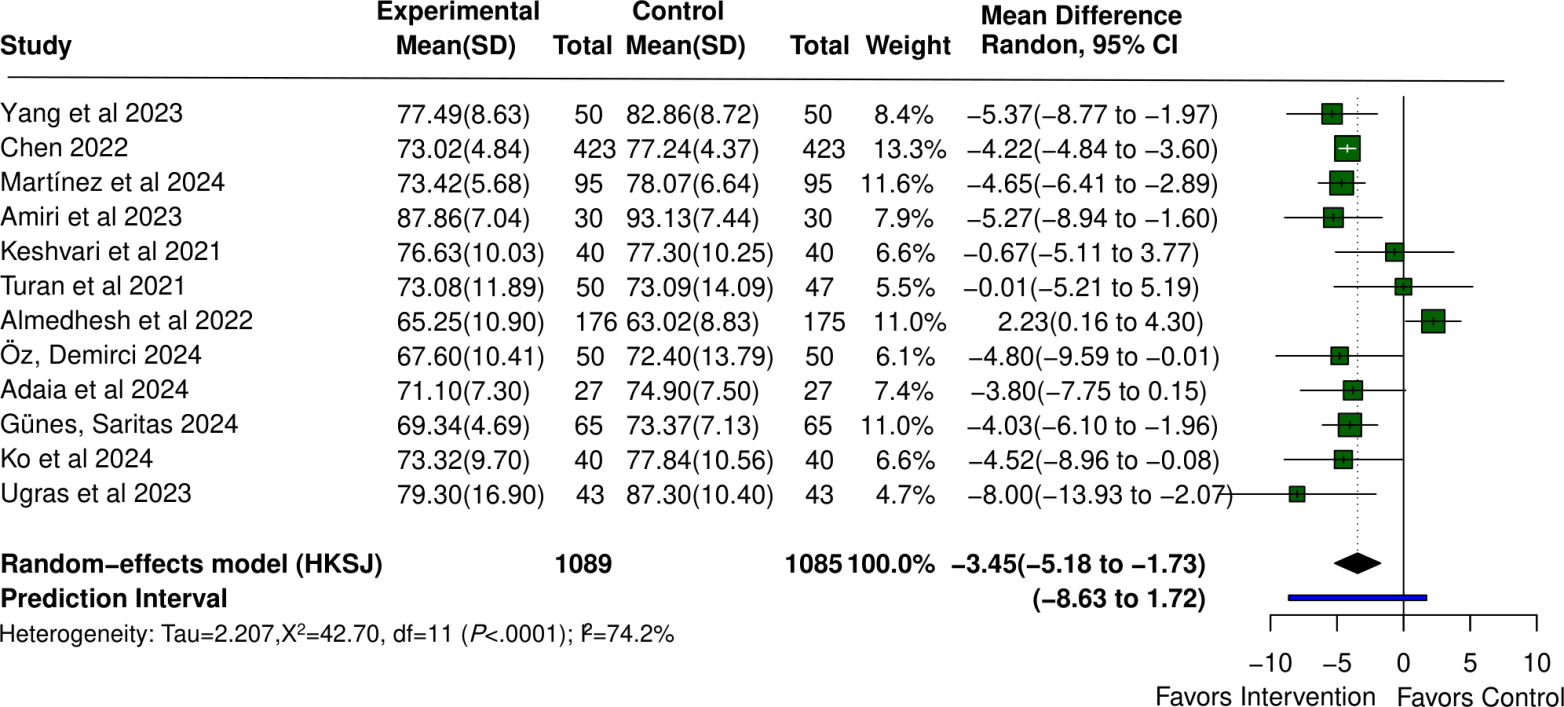
Forest plot of the mean difference for the effect of virtual reality (VR) on diastolic blood pressure in patients undergoing surgery compared with control [23,37,38,40-44,47,50,59,74].

VR also significantly reduced DBP (MD −3.45, 95% CI −5.18 to −1.73; *P*<.001). Significant heterogeneity was detected (τ²=4.870; *I*²=74.2%), with a 95% PI of −8.63 to 1.72. Forest plots for blood pressure outcomes are shown in Figure 5 [23,37,38,40-44,47,50,51,59,74] and Figure 6 [23,37,38,40-44,47,50,59,74].

### Heart Rate

**Figure 7.**
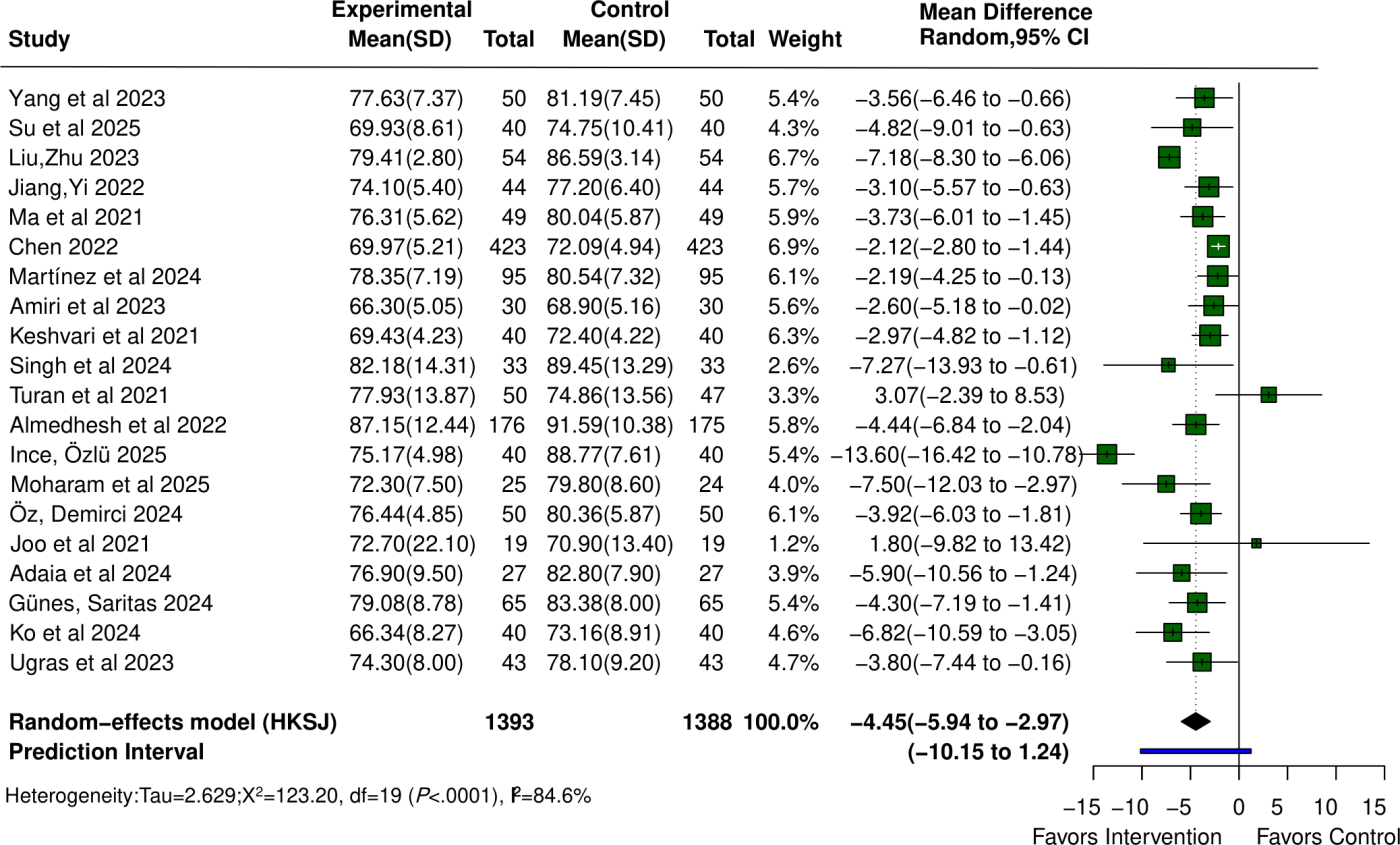
Forest plot of the mean difference for the effect of virtual reality (VR) on heart rate in patients undergoing perioperative care compared with control [23,37-44,47,50,51,55,56,59,67-70,73].

Twenty studies [23,37-44,47,50,51,55,56,59,67-70,73] reported the effect of VR on heart rate in patients undergoing perioperative care, with a total sample size of 2781 participants. The pooled effect size showed that VR intervention significantly reduced heart rate (MD −4.45, 95% CI −5.94 to −2.97; *P*<.001). Significant heterogeneity was observed across studies (Q=123.20; *P*<.001; τ=2.629; τ²=6.914; *I*²=84.6%), and the 95% PI was −10.15 to 1.24. The forest plot is provided in Figure 7 [23,37-44,47,50,51,55,56,59,67-70,73].

### Sensitivity Analysis and Small-Study Effects Assessment

A leave-one-out sensitivity analysis was performed to assess the robustness of the 5 outcome indicators: anxiety, depression, SBP, DBP, and heart rate. After sequentially removing individual studies, the pooled effect sizes were recalculated using the Hartung-Knapp-Sidik-Jonkman random-effects model. The results demonstrated that omitting any single study did not cause a directional change in the overall pooled effect sizes, and all pooled estimates remained statistically significant (*P*<.001), indicating that the meta-analysis results were highly robust. Forest plots of the sensitivity analysis are shown in Figure S1 in Multimedia Appendix 2.

Small-study effects were evaluated using funnel plots combined with the Egger linear regression test, which was performed only for outcomes with ≥10 included studies (anxiety, SBP, DBP, and heart rate):

- Anxiety: funnel plot was generally symmetric (Egger test: *t*=−0.10; *P*=.92)
- SBP: funnel plot was generally symmetric (Egger test: *t*=−0.84; *P*=.42)
- DBP: funnel plot was generally symmetric (Egger test: *t*=0.55; *P*=.60)
- Heart rate: funnel plot was generally symmetric (Egger test: *t*=−1.08; *P*=.30)

None of the Egger tests for the above outcomes were statistically significant (*P*>.05), indicating no significant small-study effects. For depression (n=7), the Egger regression test suggested asymmetry in the funnel plot (*t*=−5.68; *P*=.002). Further sensitivity verification using the trim-and-fill method showed that the pooled effect size after adjustment was consistent with the original result (SMD −1.26, 95% CI −1.71 to −0.81; *P*<.05). This suggests that the observed funnel plot asymmetry was more likely caused by heterogeneity in VR intervention content and timing across studies, rather than publication bias, further confirming the authenticity and robustness of VR in improving perioperative depression. The results are shown in Figure S2 in Multimedia Appendix 3.

## Discussion

### Principal Findings

This review aimed to comprehensively evaluate the efficacy of VR interventions on psychological emotions and physiological stress in patients undergoing perioperative care. Consistent with our initial hypotheses, the findings from 42 included RCTs confirm that, compared to conventional perioperative care, adjunctive VR interventions significantly reduce patients’ anxiety and depression levels. Furthermore, the results validate our hypothesis regarding objective physiological metrics, demonstrating that VR effectively lowers SBP and DBP while decreasing heart rate. These dual regulatory benefits offer strong, evidence-based support for VR as a safe and efficacious nonpharmacological intervention for stabilizing perioperative stress.

Subgroup analyses showed that the anxiolytic effect of VR was more pronounced in Asian populations. The 95% PI indicated that the effect of VR on depression was stable in most clinical settings, whereas its effects on anxiety, blood pressure, and heart rate were influenced by heterogeneity and might not be significant in some specific contexts. Sensitivity analyses confirmed the high robustness of the results. Assessment of small-study effects revealed no significant small-study effects for any outcome except depression.

This study demonstrated that VR technology effectively alleviates anxiety and depression in patients undergoing perioperative care, which is consistent with findings from previous relevant systematic reviews [75]. The core mechanisms underlying the psychological stress-relieving effect of VR are mainly attributed to distraction theory and information processing theory [76]. On the one hand, the perioperative environment (eg, alarms from monitoring equipment and unfamiliar instruments) serves as a direct stressor triggering anxiety and depression in patients [77]. By constructing a highly immersive 3D audio-visual environment, VR can effectively redirect patients’ limited attention, disconnect them from the negative surrounding environment, and thereby reduce the development of depressive symptoms [76,78]. On the other hand, for anticipatory anxiety induced by the unknown nature of surgery, preoperative full-process scenario simulation via VR (eg, first-person experience of anesthesia induction) can significantly reduce information asymmetry between physicians and patients [20]. When patients develop a clear understanding of the treatment procedure and gain a sense of control, their inner uncertainty and helplessness are substantially diminished [49].

Furthermore, subgroup analyses revealed an important finding: the anxiolytic effect of VR intervention was more significant among patients in Asian regions. This is consistent with the cross-cultural findings reported by Streuli et al [79]. Possible reasons include that in Asian cultures, patients tend to express negative emotions less directly; VR, as an implicit and nonconfrontational intervention, better matches the psychological acceptance of this population [80]. Most studies [23,35,37,39,47-49,51,56,58,66] conducted in Asian regions used VR scenes featuring natural landscapes, which may enhance patients’ immersive experience and adherence to interventions. In addition, intraoperative interventions exhibited extremely low heterogeneity. This may be attributed to the fact that patients under local or neuraxial anesthesia during surgery are more susceptible to distraction by VR content, thus reducing between-study heterogeneity [39,41].

At the physiological level, this review confirmed that VR effectively reduces blood pressure and heart rate, findings highly consistent with those of the single-center clinical trial by Ugras et al [23]. Patients undergoing perioperative care are under severe psychological stress due to fear and uncertainty, which directly activates the hypothalamic-pituitary-adrenal axis and sympathetic nervous system, leading to massive catecholamine release, followed by tachycardia, peripheral vasoconstriction, and hypertension [81]. Through its powerful psychological soothing effects, VR interrupts this psychosomatic stress cycle. Functional magnetic resonance imaging studies have confirmed that immersive VR experiences not only regulate the balance of the autonomic nervous system (ie, reducing sympathetic activity and enhancing parasympathetic tone) but also significantly decrease activation in brain regions involved in pain and emotional processing, such as the anterior cingulate cortex, insula, and somatosensory cortex [82]. Such inhibitory effects at the central neural level represent the fundamental physiological basis for VR to stabilize perioperative hemodynamic parameters.

The findings of this review carry important implications for clinical nursing practice. First, as key providers of perioperative care, health care professionals act as a bridge between technology and patients in VR interventions [83]. They are required not only to operate the equipment but also to assess patients’ digital literacy, medical history, and preferences before intervention to select the most suitable VR content (eg, relaxing natural scenes or cognitive educational videos). During intervention implementation, they should closely monitor changes in patients’ heart rate and blood pressure, which serve as objective indicators for dynamically adjusting the intervention protocol [84].

Second, although the clinical efficacy of VR technology has been confirmed, its widespread implementation still faces cost-effectiveness considerations. The initial investments in hardware procurement, software customization, and operational training are relatively high [85]. Nevertheless, several health economic studies on digital therapeutics have demonstrated that, in the long run, VR interventions help reduce postoperative analgesic requirements, shorten anesthesia recovery time, and decrease overall length of hospital stay, thus yielding significant cost-effectiveness [86,87]. Therefore, hospital administrators should focus on the overall health economic value when introducing this technology.

### Innovations and Limitations

Regarding the review process itself, no significant process-specific limitations were identified. This review is the first to comprehensively evaluate VR interventions in the perioperative setting by incorporating anxiety, depression, and objective hemodynamic parameters (blood pressure and heart rate). It overcomes the limitation of previous studies [24-26] that were only focused on anxiety alone and more comprehensively reveals the intervention effect of VR on both psychological and physiological stress during the perioperative period, which is consistent with the pathophysiological characteristics of perioperative stress [3,81]. Through subgroup analyses, the impacts of region and intervention timing on VR efficacy were identified, providing evidence for the clinical design of personalized VR intervention protocols—for example, VR can be prioritized for Asian patients, and standardized intraoperative VR programs can be adopted to reduce heterogeneity.

This review has several limitations that should be considered when interpreting the results. First, substantial clinical heterogeneity was observed across the included studies regarding types of VR devices (eg, head-mounted display models), intervention content (relaxation-based vs education-based), exposure frequency, and specific surgical types [23,49], which may have reduced the precision of the pooled effect sizes to some extent. Second, owing to the inherent physical characteristics of VR interventions, it was difficult for the included studies to achieve genuine double-blinding of patients and implementing nurses [20,75], which inevitably introduced risks of performance bias and measurement bias. Third, the number of studies included on depression was relatively small [34,35,61,63,64,69,71], leading to low-quality evidence. Therefore, the corresponding results should be interpreted with caution, and more high-quality studies focusing on VR interventions for perioperative depression are needed in the future.

### Future Research Directions

Future studies should standardize the reporting of VR devices, intervention content, and exposure parameters and clarify the impact of different surgical types and intervention protocols on pooled effect sizes. To address bias arising from blinding limitations, it is recommended to strictly implement outcome-assessor blinding and include objective physiological indicators to ensure reliable results. Furthermore, researchers should conduct multicenter, large-sample randomized trials and extend follow-up periods to build high-quality evidence regarding the long-term efficacy of VR.

### Conclusion

This systematic review and meta-analysis demonstrate that VR serves as a safe and effective adjunctive intervention that exhibits a dual regulatory mechanism in perioperative adult patients. Based on the 95% CIs, VR significantly alleviates average psychological distress (anxiety and depression) and stabilizes physiological indicators (SBP, DBP, and heart rate). However, while the CIs confirm a robust average benefit, the wide 95% PIs indicate that, due to substantial clinical heterogeneity, the true effect of VR for an individual patient in future clinical settings may vary widely. Furthermore, these findings should be interpreted cautiously; due to the inherent difficulty of double-blinding in VR interventions and the diverse VR contents and devices used, the GRADE assessment indicates a moderate-to-low certainty of evidence across all outcomes.

The innovation of this study lies in its comprehensive evaluation framework. Unlike existing systematic reviews that are predominantly limited to a single psychological metric (eg, anxiety alone) or focused on pediatric populations, this review innovatively integrates both psychological and objective physiological dimensions to comprehensively assess the perioperative psychosomatic stress response. Contributing to the field, this multidimensional assessment clarifies the dual regulatory mechanism of VR in mitigating perioperative stress. In terms of real-world implications, this review provides valuable, evidence-based guidance for health care professionals to implement personalized digital nonpharmacological interventions—such as prioritizing VR use for Asian perioperative populations, where the anxiolytic effect is notably pronounced. Ultimately, integrating VR into clinical practice can reduce reliance on pharmacological interventions, improve the patient care experience, and enhance the quality of postoperative recovery. Future multicenter, large-sample RCTs with standardized protocols and strict blinding are warranted to further elevate the quality of evidence.

## Supplementary material

10.2196/81799 Multimedia Appendix 1 Literature search formula.

10.2196/81799 Multimedia Appendix 2 Sensitivity analysis results for all outcomes.

10.2196/81799 Multimedia Appendix 3 Funnel plots for publication bias assessment.

10.2196/81799 Checklist 1 PRISMA-S checklist.

10.2196/81799 Checklist 2 PRISMA 2020 checklist.

## Abbreviations

CBM: Chinese Biomedical Literature Database
CNKI: China National Knowledge Infrastructure
DBP: diastolic blood pressure
GRADE: Grading of Recommendations, Assessment, Development, and Evaluation
MD: mean difference
PI: prediction interval
PRISMA: Preferred Reporting Items for Systematic Reviews and Meta-Analysis
PRISMA-S: Preferred Reporting Items for Systematic Reviews and Meta-Analysis–Search
PROSPERO: International Prospective Register of Systematic Reviews
RCT: randomized controlled trial
SBP: systolic blood pressure
SMD: standardized mean difference
VIP: China Science and Technology Journal Database
VR: virtual reality
