# Supplementary material for: Effectiveness of Virtual Reality Interventions on Perioperative Anxiety, Depression, Blood Pressure, and Heart Rate: Systematic Review and Meta-Analysis of Randomized Controlled Trials
Source: JMIR Serious Games. 2026 May 19;14:e81799. doi: 10.2196/81799 (PMC13188064; doi:10.2196/81799)
Supplement: Multimedia Appendix 1 [file games-v14-e81799-s001.pdf]

Search terms used

CNKI database Search Strategy Table				
**Search Number**	**Query**	**Filters**	**Search Details**	**Results**
1	(虚拟现实 + VR + 虚拟实境 + 沉浸式虚拟技术) AND (围术期 + 手术期 + 术前 + 术中 + 术后 + 手术患者 + 外科患者) AND (焦虑 + 抑郁 + 血压 + 心率 + 收缩压 + 舒张压 + 心率变异性 + 负性情绪)	No screening criteria applied	Advanced Search - Subject field	82
2	(虚拟现实 + VR + 虚拟实境 + 沉浸式虚拟技术) AND (围术期 + 手术期 + 术前 + 术中 + 术后 + 手术患者 + 外科患者) AND (焦虑 + 抑郁 + 血压 + 心率 + 收缩压 + 舒张压 + 心率变异性 + 负性情绪)	1. Time range: 2000-01-01 to 2026-03-06	Advanced Search - Subject field	82
3	(虚拟现实 + VR + 虚拟实境 + 沉浸式虚拟技术) AND (围术期 + 手术期 + 术前 + 术中 + 术后 + 手术患者 + 外科患者) AND (焦虑 + 抑郁 + 血压 + 心率 + 收缩压 + 舒张压 + 心率变异性 + 负性情绪)	1. Time range: 2000-01-01 to 2026-03-06.<br>2. Document type: journal articles, dissertations, conference papers	Advanced Search - Subject field	81

Wan Fang database Search Strategy Table				
**Search Number**	**Query**	**Filters**	**Search Details**	**Results**
1	(虚拟现实 OR VR OR 虚拟实境 OR 沉浸式虚拟技术) AND (围术期 OR 手术期 OR 术前 OR 术中 OR 术后 OR 手术患者 OR 外科患者) AND (焦虑 OR 抑郁 OR 血压 OR 心率 OR 收缩压 OR 舒张压 OR 心率变异性 OR 负性情绪)	No screening criteria applied	Advanced Search - Subject field	1084
2	(虚拟现实 OR VR OR 虚拟实境 OR 沉浸式虚拟技术) AND (围术期 OR 手术期 OR 术前 OR 术中 OR 术后 OR 手术患者 OR 外科患者) AND (焦虑 OR 抑郁 OR 血压 OR 心率 OR 收缩压 OR 舒张压 OR 心率变异性 OR 负性情绪)	1. Time range: 2000-01-01 to 2026-03-06	Advanced Search - Subject field	1068
3	(虚拟现实 OR VR OR 虚拟实境 OR 沉浸式虚拟技术) AND (围术期 OR 手术期 OR 术前 OR 术中 OR 术后 OR 手术患者 OR 外科患者) AND (焦虑 OR 抑郁 OR 血压 OR 心率 OR 收缩压 OR 舒张压 OR 心率变异性 OR 负性情绪)	1. Time range: 2000-01-01 to 2026-03-06.<br><br>2. Document type: journal articles, dissertations, conference papers	Advanced Search - Subject field	905

VIP database Search Strategy Table

Search Number	Query	Filters	Search Details	Results
1	(虚拟现实 OR VR OR 虚拟实境 OR 沉浸式虚拟技术) AND (围术期 OR 手术期 OR 术前 OR 术中 OR 术后 OR 手术患者 OR 外科患者) AND (焦虑 OR 抑郁 OR 血压 OR 心率 OR 收缩压 OR 舒张压 OR 心率变异性 OR 负性情绪)	No screening criteria applied	Advanced Search - Any field	308

SinoMed Search Strategy Table

Search Number	Query	Filters	Search Details	Results
1	(主题=虚拟现实 OR 虚拟现实 OR VR OR 沉浸式虚拟技术) AND（主题=围术期 OR 手术期 OR 术前 OR 术中 OR 术后 OR 手术患者) AND (主题=焦虑 OR 抑郁 OR 血压 OR 心率 OR 收缩压 OR 舒张压 OR 心率变异性)	No screening criteria applied	Advanced Search - Subject field	1014
2	(主题=虚拟现实 OR 虚拟现实 OR VR OR 沉浸式虚拟技术) AND（主题=围术期 OR 手术期 OR 术前 OR 术中 OR 术后 OR 手术患者) AND (主题=焦虑 OR 抑郁 OR 血压 OR 心率 OR 收缩压 OR 舒张压 OR 心率变异性)	1. Time range: 2000-01-01 to 2026-03-06	Advanced Search - Subject field	1004

3	(主题=虚拟现实 OR 虚拟现实 OR VR OR 沉浸式虚拟技术) AND（主题=围术期 OR 手术期 OR 术前 OR 术中 OR 术后 OR 手术患者) AND (主题=焦虑 OR 抑郁 OR 血压 OR 心率 OR 收缩压 OR 舒张压 OR 心率变异性)	1. Time range: 2000-01-01 to 2026-03-06.<br>2. Document type: journal articles, dissertations, conference papers	Advanced Search - Subject field	561
4	(主题=虚拟现实 OR 虚拟现实 OR VR OR 沉浸式虚拟技术) AND（主题=围术期 OR 手术期 OR 术前 OR 术中 OR 术后 OR 手术患者) AND (主题=焦虑 OR 抑郁 OR 血压 OR 心率 OR 收缩压 OR 舒张压 OR 心率变异性)	1. Time range: 2000-01-01 to 2026-03-06.<br>2. Document type: journal articles, dissertations, conference papers<br>3. Research method：randomized controlled trial	Advanced Search - Subject field	71
**Embase Search Strategy Table**				
1	("virtual reality"/exp OR VR OR "immersive virtual reality" OR "virtual reality technology") AND ("perioperative period"/exp OR "surgical patient"/exp OR preoperative OR intraoperative OR postoperative) AND ("anxiety"/exp OR "depression"/exp OR "blood pressure"/exp	No screening criteria applied	Advanced Search, combining Emtree terms and free words	1168

	OR "heart rate"/exp OR BP OR SBP OR DBP OR HR OR HRV OR "heart rate variability")			
2	("virtual reality"/exp OR VR OR "immersive virtual reality" OR "virtual reality technology") AND ("perioperative period"/exp OR "surgical patient"/exp OR preoperative OR intraoperative OR postoperative) AND ("anxiety"/exp OR "depression"/exp OR "blood pressure"/exp OR "heart rate"/exp OR BP OR SBP OR DBP OR HR OR HRV OR "heart rate variability")	1. Time range: 2000-01-01 to 2026-03-06.	Advanced Search, combining Emtree terms and free words	1141
3	("virtual reality"/exp OR VR OR "immersive virtual reality" OR "virtual reality technology") AND ("perioperative period"/exp OR "surgical patient"/exp OR preoperative OR intraoperative OR postoperative) AND ("anxiety"/exp OR "depression"/exp OR "blood pressure"/exp OR "heart rate"/exp OR	1. Time range: 2000-01-01 to 2026-03-06.<br>2. Publication type: articles, clinical trail, conference papers	Advanced Search, combining Emtree terms and free words	768

	BP OR SBP OR DBP OR HR OR HRV OR "heart rate variability")			
4	("virtual reality"/exp OR VR OR "immersive virtual reality" OR "virtual reality technology") AND ("perioperative period"/exp OR "surgical patient"/exp OR preoperative OR intraoperative OR postoperative) AND ("anxiety"/exp OR "depression"/exp OR "blood pressure"/exp OR "heart rate"/exp OR BP OR SBP OR DBP OR HR OR HRV OR "heart rate variability")	1. Time range: 2000-01-01 to 2026-03-06.<br>2. Publication type: articles, clinical trail, conference papers<br>3. Research method：randomized controlled trial	Advanced Search, combining Emtree terms and free words	355
**Pubmed Search Strategy Table**				
1	((("Virtual Reality"[MeSH Terms] OR "Virtual Reality"[All Fields] OR VR[All Fields] OR "immersive virtual reality"[All Fields] OR "virtual reality technology"[All Fields]) AND ("Perioperative Period"[MeSH Terms] OR "perioperative"[All Fields] OR "preoperative"[All Fields] OR "intraoperative"[All	No screening criteria applied	PubMed homepage + MeSH Database, combining MeSH terms and field tags	423

	Fields] OR "postoperative"[All Fields] OR "surgical patient"[All Fields] OR "surgery patient"[All Fields])) AND ("Anxiety"[MeSH Terms] OR "Depression"[MeSH Terms] OR "Blood Pressure"[MeSH Terms] OR "Heart Rate"[MeSH Terms] OR "anxiety"[All Fields] OR "depression"[All Fields] OR "BP"[All Fields] OR "HR"[All Fields] OR "SBP"[All Fields] OR "DBP"[All Fields] OR "heart rate variability"[All Fields] OR "HRV"[All Fields]))			
2	((("Virtual Reality"[MeSH Terms] OR "Virtual Reality"[All Fields] OR VR[All Fields] OR "immersive virtual reality"[All Fields] OR "virtual reality technology"[All Fields]) AND ("Perioperative Period"[MeSH Terms] OR "perioperative"[All Fields] OR "preoperative"[All Fields] OR "intraoperative"[All Fields] OR "postoperative"[All	1. Time range: 2000-01-01 to 2026-03-06.	PubMed homepage + MeSH Database, combining MeSH terms and field tags	415

	Fields] OR "surgical patient"[All Fields] OR "surgery patient"[All Fields])) AND ("Anxiety"[MeSH Terms] OR "Depression"[MeSH Terms] OR "Blood Pressure"[MeSH Terms] OR "Heart Rate"[MeSH Terms] OR "anxiety"[All Fields] OR "depression"[All Fields] OR "BP"[All Fields] OR "HR"[All Fields] OR "SBP"[All Fields] OR "DBP"[All Fields] OR "heart rate variability"[All Fields] OR "HRV"[All Fields]))			
3	((("Virtual Reality"[MeSH Terms] OR "Virtual Reality"[All Fields] OR VR[All Fields] OR "immersive virtual reality"[All Fields] OR "virtual reality technology"[All Fields]) AND ("Perioperative Period"[MeSH Terms] OR "perioperative"[All Fields] OR "preoperative"[All Fields] OR "intraoperative"[All Fields] OR "postoperative"[All Fields] OR "surgical patient"[All Fields] OR	1. Time range: 2000-01-01 to 2026-03-06.<br>2. Artical type:randomized controlled trial	PubMed homepage + MeSH Database, combining MeSH terms and field tags	109

	"surgery patient"[All Fields])) AND ("Anxiety"[MeSH Terms] OR "Depression"[MeSH Terms] OR "Blood Pressure"[MeSH Terms] OR "Heart Rate"[MeSH Terms] OR "anxiety"[All Fields] OR "depression"[All Fields] OR "BP"[All Fields] OR "HR"[All Fields] OR "SBP"[All Fields] OR "DBP"[All Fields] OR "heart rate variability"[All Fields] OR "HRV"[All Fields]))			
**Web of Science Search Strategy Table**				
1	TS=(virtual reality OR VR OR immersive virtual reality OR virtual reality technology) AND TS=(perioperative OR preoperative OR intraoperative OR postoperative OR surgical patient OR surgical population) AND TS=(anxiety OR depression OR blood pressure OR BP OR SBP OR DBP OR heart rate OR HR OR HRV)	No screening criteria applied	Core Collection - Advanced Search - Topic (TS) field	416
2	TS=(virtual reality OR VR OR immersive virtual reality OR virtual reality technology) AND	1. Publication type: articles, clinical trail, conference papers	Core Collection - Advanced Search - Topic	403

	TS=(perioperative OR preoperative OR intraoperative OR postoperative OR surgical patient OR surgical population) AND TS=(anxiety OR depression OR blood pressure OR BP OR SBP OR DBP OR heart rate OR HR OR HRV)		(TS) field	
3	TS=(virtual reality OR VR OR immersive virtual reality OR virtual reality technology) AND TS=(perioperative OR preoperative OR intraoperative OR postoperative OR surgical patient OR surgical population) AND TS=(anxiety OR depression OR blood pressure OR BP OR SBP OR DBP OR heart rate OR HR OR HRV)	1. Publication type: articles, clinical trail, conference papers<br>2. Language:English,Chinese	Core Collection - Advanced Search - Topic (TS) field	402
**Scopus Search Strategy Table**				
1	ALL(virtual reality OR VR OR immersive virtual reality OR virtual reality technology) AND ALL(perioperative OR preoperative OR intraoperative OR postoperative OR surgical patient OR surgical population) AND	No screening criteria applied	Advanced Search, using ALL() syntax	1400

	ALL(anxiety OR depression OR blood pressure OR BP OR SBP OR DBP OR heart rate OR HR OR HRV)			
2	ALL(virtual reality OR VR OR immersive virtual reality OR virtual reality technology) AND ALL(perioperative OR preoperative OR intraoperative OR postoperative OR surgical patient OR surgical population) AND ALL(anxiety OR depression OR blood pressure OR BP OR SBP OR DBP OR heart rate OR HR OR HRV)	1. Time range: 2000-01-01 to 2026-03-06.	Advanced Search, using ALL() syntax	1399
3	ALL(virtual reality OR VR OR immersive virtual reality OR virtual reality technology) AND ALL(perioperative OR preoperative OR intraoperative OR postoperative OR surgical patient OR surgical population) AND ALL(anxiety OR depression OR blood pressure OR BP OR SBP OR DBP OR heart rate OR HR OR HRV)	1. Time range: 2000-01-01 to 2026-03-06.<br>2. Publication type: articles, clinical trail, conference papers	Advanced Search, using ALL() syntax	396

The Cochrane Library Search Strategy Table

1	(virtual reality OR VR OR immersive virtual reality) AND (perioperative OR preoperative OR intraoperative OR postoperative) AND (anxiety OR depression OR blood pressure OR heart rate OR BP OR HR)	No screening criteria applied	CENTRAL - Advanced Search, adapted to PICO framework	591
2	(virtual reality OR VR OR immersive virtual reality) AND (perioperative OR preoperative OR intraoperative OR postoperative) AND (anxiety OR depression OR blood pressure OR heart rate OR BP OR HR)	1. Publication type: articles, clinical trail, conference papers	CENTRAL - Advanced Search, adapted to PICO framework	562
3	(virtual reality OR VR OR immersive virtual reality) AND (perioperative OR preoperative OR intraoperative OR postoperative) AND (anxiety OR depression OR blood pressure OR heart rate OR BP OR HR)	1. Publication type: articles, clinical trail, conference papers<br>2. Language:English,Chinese	CENTRAL - Advanced Search, adapted to PICO framework	559
