# Supplementary figures and images for: Effectiveness of Virtual Reality Interventions on Perioperative Anxiety, Depression, Blood Pressure, and Heart Rate: Systematic Review and Meta-Analysis of Randomized Controlled Trials
Source: JMIR Serious Games. 2026 May 19;14:e81799. doi: 10.2196/81799 (PMC13188064; doi:10.2196/81799)

## (a)Anxiety

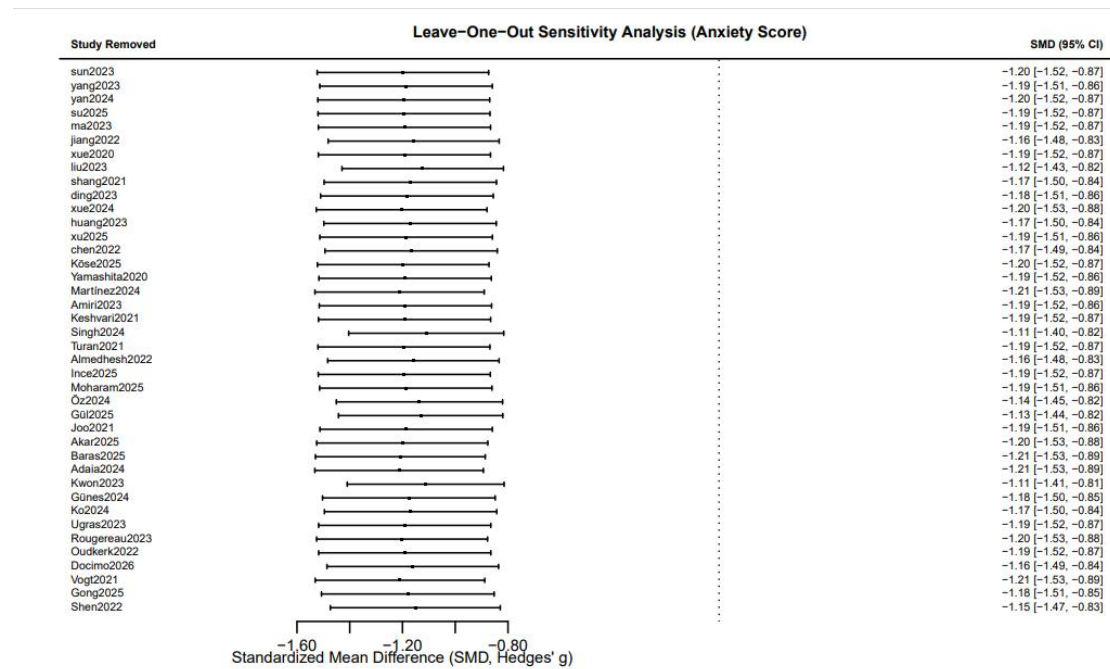

## (b)Depression

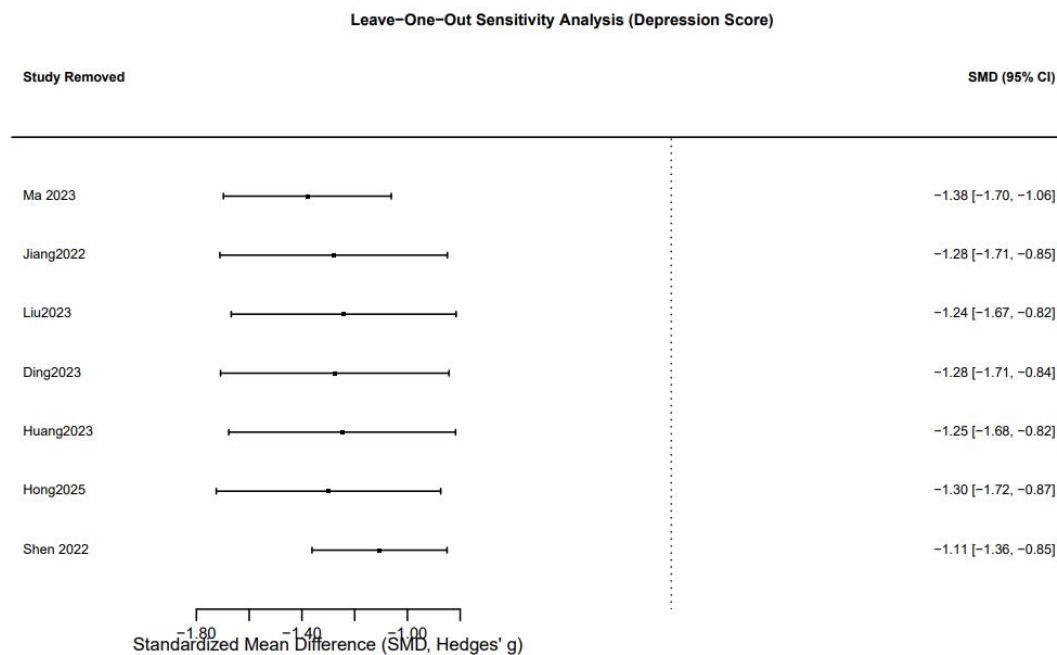

## (c)Systolic Blood Pressure

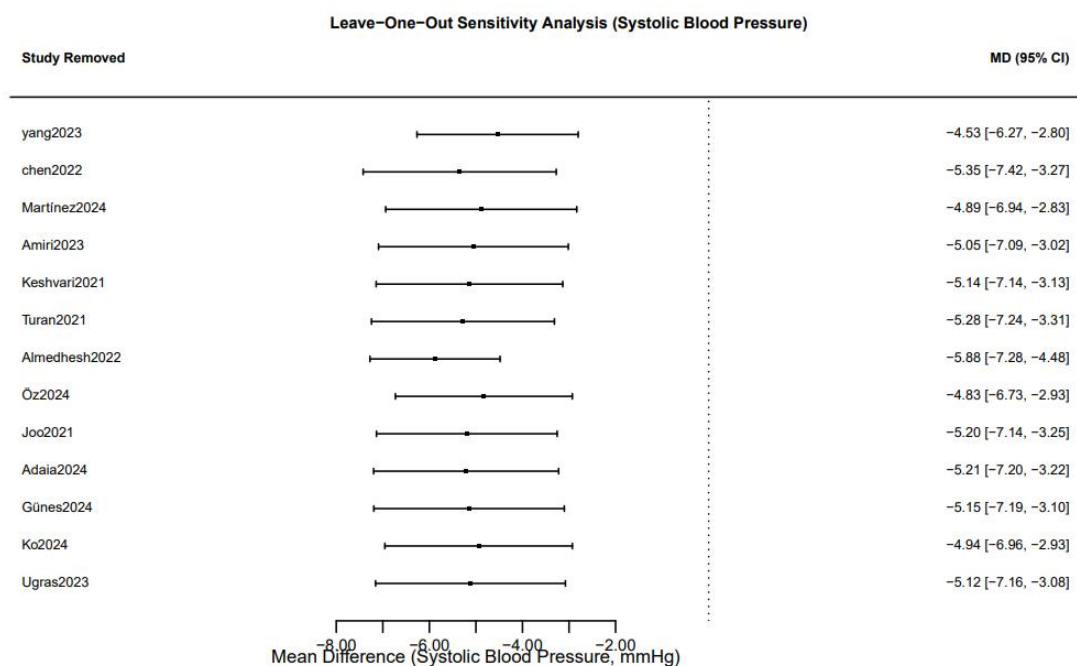

#### (d) Diastolic Blood Pressure

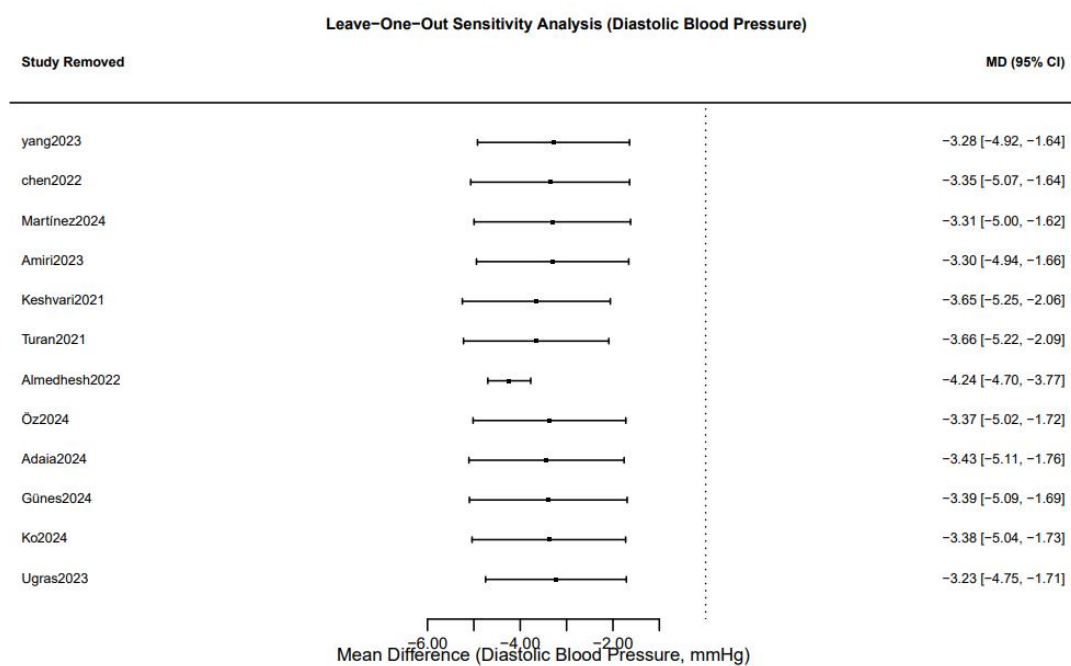

#### (e) Heart Rate

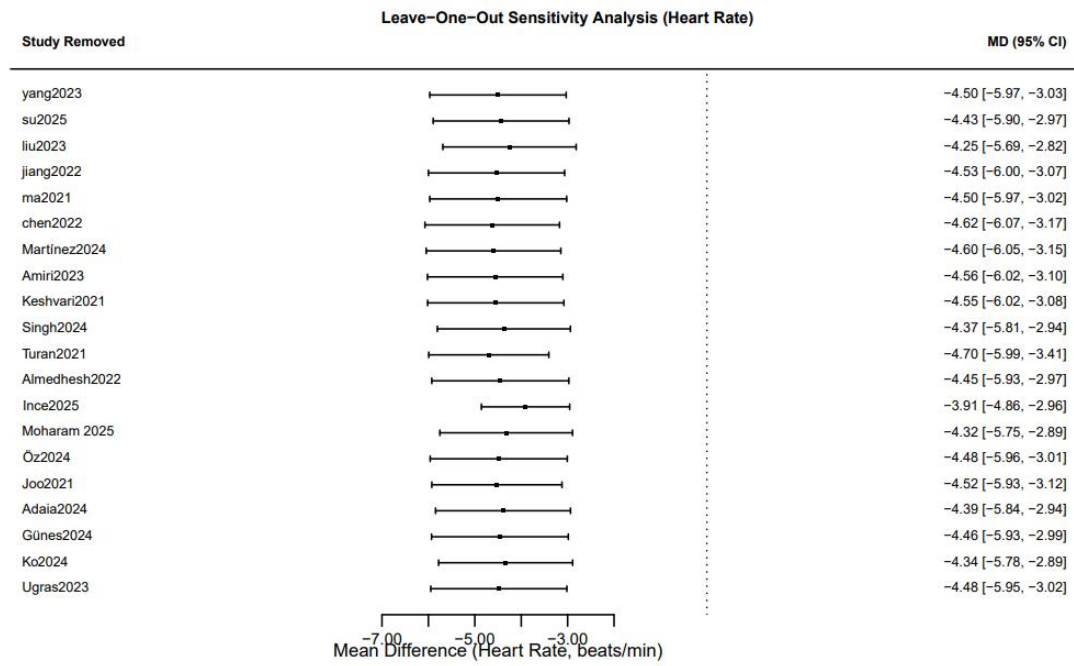

Supplement: Multimedia Appendix 2 [file games-v14-e81799-s002.pdf]

(a)Anxiety

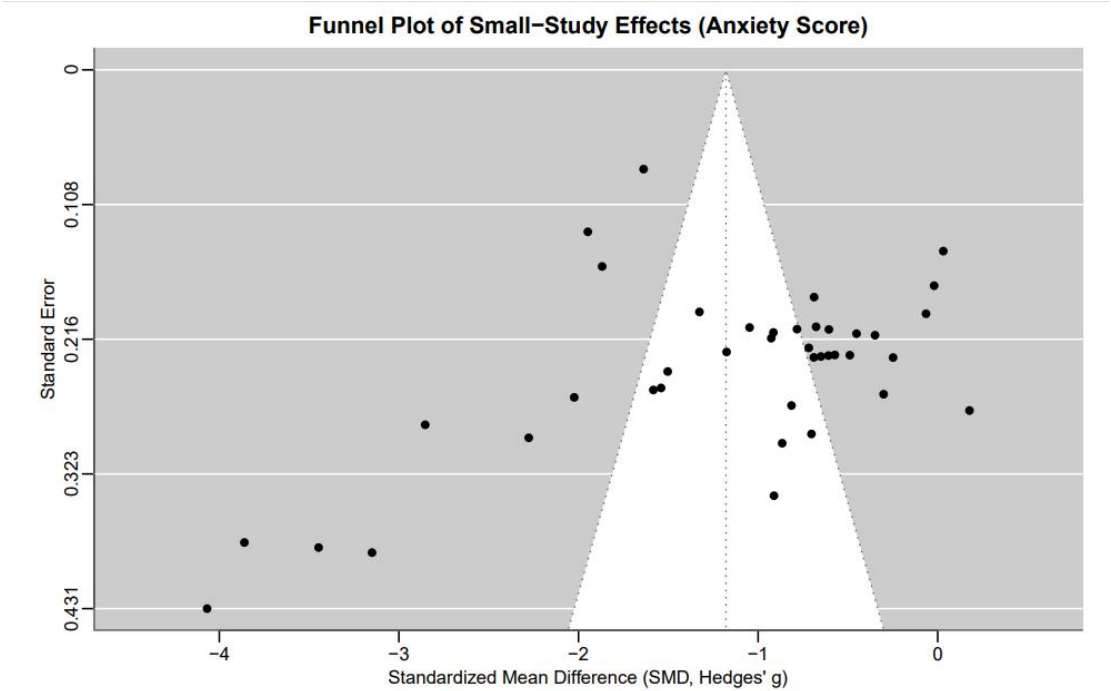

(b) Depression

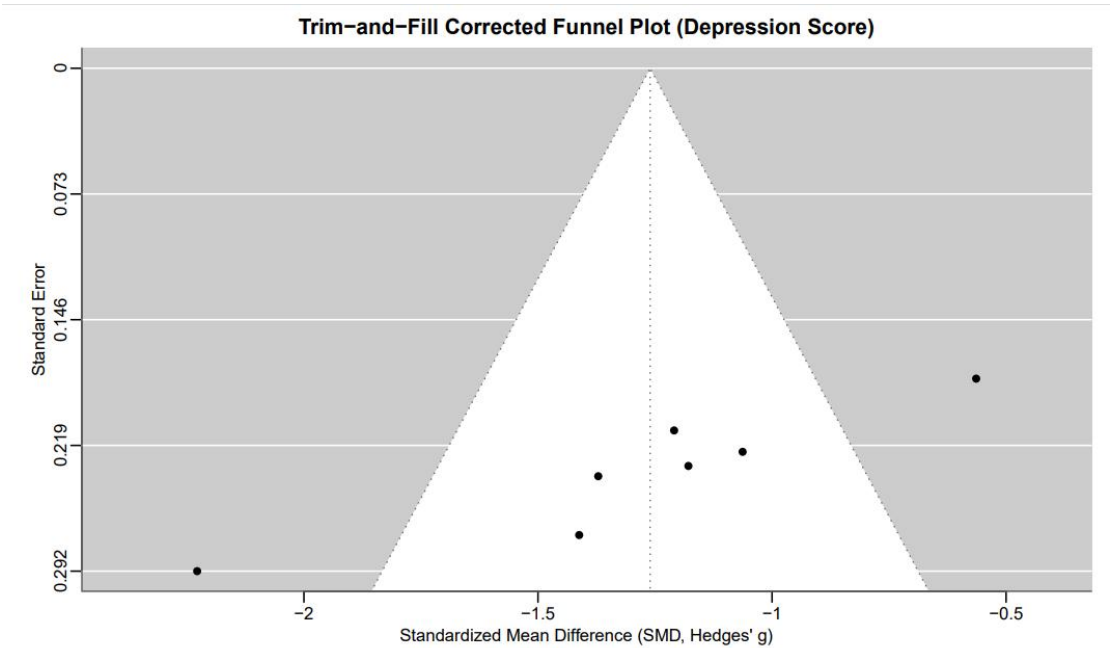

(c)Systolic Blood Pressure

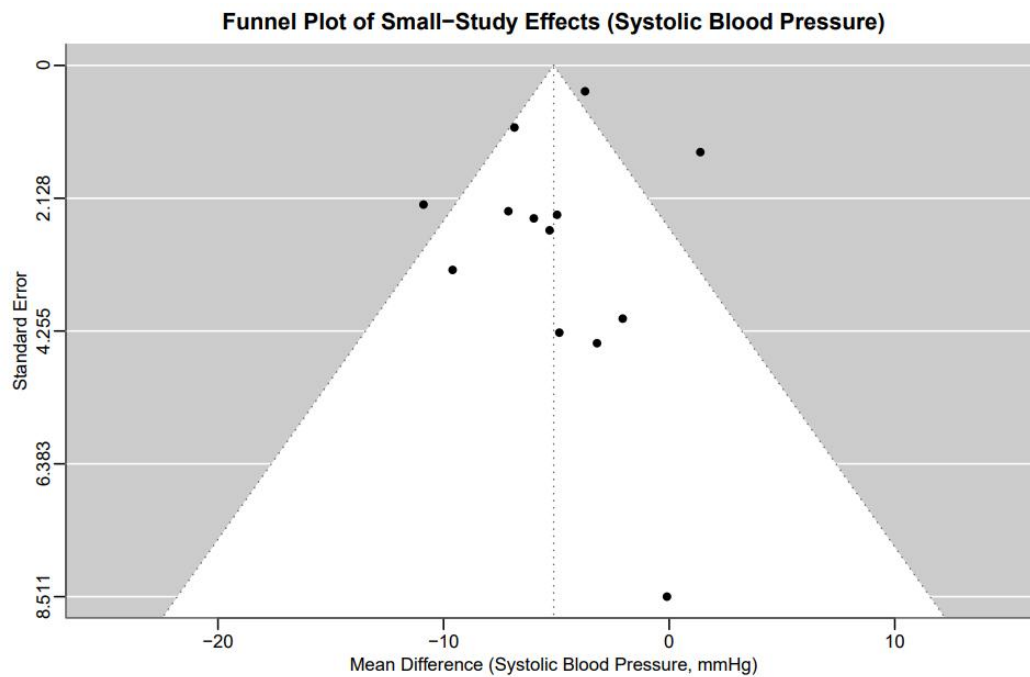

(d) Diastolic Blood Pressure

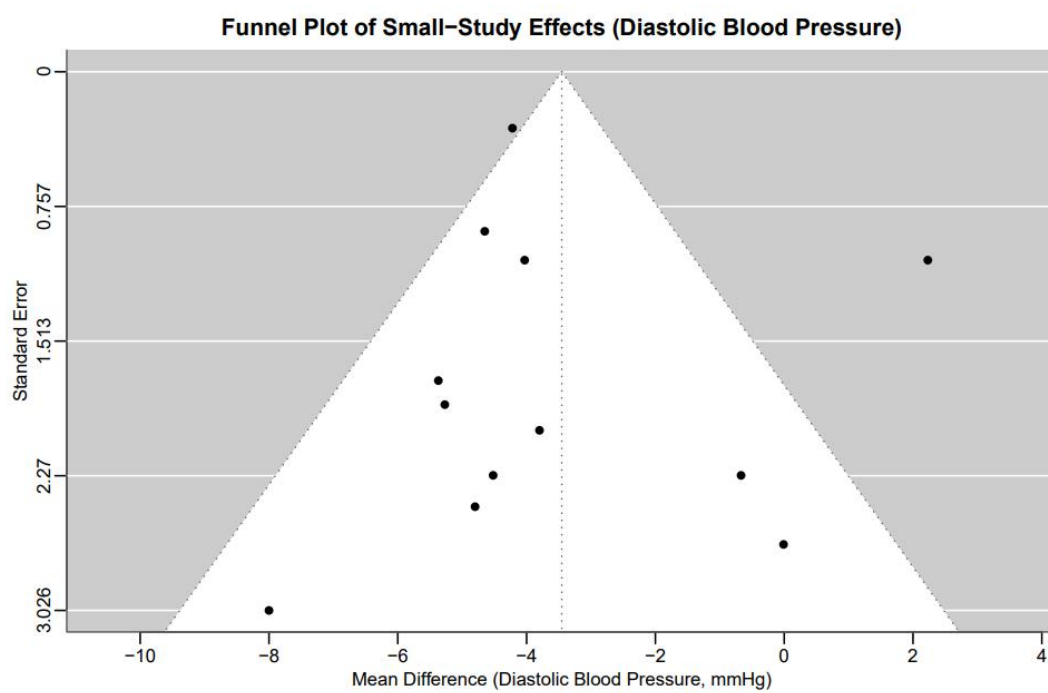

(e) Heart Rate

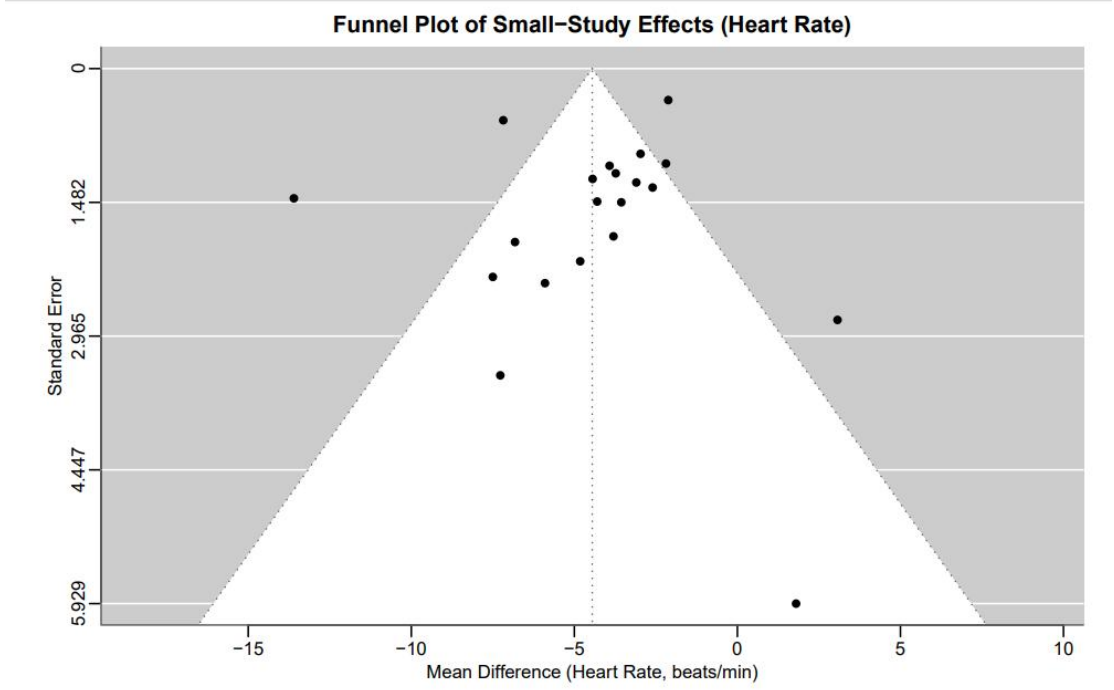

Supplement: Multimedia Appendix 3 [file games-v14-e81799-s003.pdf]
